# Direct microwave energy input on a single cation for outstanding selective catalysis

**DOI:** 10.1126/sciadv.adi1744

**Published:** 2023-08-18

**Authors:** Fuminao Kishimoto, Tatsushi Yoshioka, Ryo Ishibashi, Hiroki Yamada, Koki Muraoka, Hiroki Taniguchi, Toru Wakihara, Kazuhiro Takanabe

**Affiliations:** ^1^ Department of Chemical System Engineering, School of Engineering, The University of Tokyo, 7-3-1 Hongo, Bunkyo-ku, Tokyo 113-8656, Japan.; ^2^ Japan Synchrotron Radiation Research Institute, SPring–8, 1-1-1 Kouto, Sayo-cho, Sayo-gun, Hyogo 679-5198, Japan.; ^3^ Department of Physics, Nagoya University, Nagoya 464-8602, Japan.; ^4^ Institute of Engineering Innovation, The University of Tokyo, 2-11-16 Yayoi, Bunkyo-ku, Tokyo 113-8656, Japan.

## Abstract

Microwave (MW)–driven catalytic systems are attracting attention not only as an aggressive electrification strategy of the chemical industry but also as creating a unique catalytic reaction field that conventional equilibrium heating cannot achieve. This study unlocked direct and selective heating of single alkali metal cations in the pores of aluminosilicate zeolites under MW. Selectively heated Cs^+^ cations in FAU zeolite exhibited selective CH_4_ combustion performance, that is, CO*_x_* generation at the heated Cs^+^ cations selectively occurred while side reactions in the low-temperature gas phase were suppressed. The Cs-O pair distribution function revealed by synchrotron-based in situ x-ray total scattering gave us direct evidence of peculiar displacement induced by MW, which was consistent with the results of molecular dynamics simulation mimicking MW heating. The concept of selective monoatomic heating by MW is expected to open a next stage in “microwave catalysis” science by providing physicochemical insights into “microwave effects.”

## Teaser

Atomic-scale energy concentration by microwaves was successfully achieved to remotely control the selectivity of catalysis.

## INTRODUCTION

To develop green chemical industries, it is important to design catalytic chemical conversion systems while avoiding undesired side reactions and ensuring that only desired reactions proceed selectively (*1*). Ultimately, high selectivity is attained without altering the catalyst design if we can create a nonequilibrium field that gives energy only to the catalytically active sites where the desired molecular activation proceeds and no energy to the other active sites. Microwaves (MWs), i.e., electromagnetic waves in the band between 0.3 and 300 GHz, enable us to selectively heat dielectric, magnetic, or conductive materials and moieties (*2*, *3*), thereby providing innovative material synthesis methods (*4*), enhanced regioselective reactions (*5*), and accelerated and selective heterogeneous catalysis (*6*–*8*). Moreover, thanks to the rapid heating characteristics of MW, electrified chemical systems driven by MW are compatible with the intermittent nature of renewable energies. Consequently, many researchers are concentrating on the development of efficient and environmentally friendly chemical conversion systems, e.g., a highly efficient MW-driven catalytic plastic splitting system to produce hydrogen and high-value carbons (*9*) and water electrolysis for hydrogen production triggered by MW-induced redox activation of CeO_2_-based materials at a much lower temperature (<250°C) (*10*). These highly efficient systems cannot be achieved by conventional heating (CH) methods based on heat transfer at the interface of the heating medium (e.g., steam and electric heater) and heating object.

The main feature of MW heating expected for such an efficient chemical conversion system is “selective heating” of the catalytically active sites or just at interfaces (*11*). Ramirez *et al.* (*12*) demonstrated that applying the localized energy on a catalyst-support (SiC monolith) ensemble induced by MW allowed the catalyst to heat hotter than the surrounding gas, achieving an acceleration of the desired heterogeneous reaction and a deceleration of the undesired homogeneous reaction. The research group further investigated the formation of temperature gradients of several hundred degrees or more on the millimeter scale between the catalyst bed and the gas by in situ temperature evaluation using a microscopic infrared (IR) camera (*13*–*16*). In more microscopic insights, nanometer-scale temperature gradients with approximately 100° were demonstrated for platinum nanoparticles on SiO_2_ support because the metallic Pt nanoparticles can be effectively heated by MW while SiO_2_ support was inert (*17*).

MW can induce the vibrational motion of ions and rotational motion of molecules and can increase the kinetic energy of those single atoms and molecules, ultimately creating, for example, a temperature gradient on the atomic scale. Note here that the term “temperature” as usually defined in the equilibrium system of standard statistical thermodynamics was used for a nonequilibrium temperature field at the atomic level (nonequilibrium temperatures and local kinetic energy are not discussed here) (*18*, *19*). Auerbach and colleagues (*20*, *21*) demonstrated that MW can induce higher translational, rotational, and vibrational kinetic energy of a single methanol molecule incorporated in siliceous zeolite pores than that of the Si-O framework of the host zeolites. Beyond the local high kinetic energy of a single molecule, selective heating of a single metal cation in the zeolite under MW has been predicted (*22*, *23*). Reported dielectric spectra of metal cation–incorporated zeolite showed that these metal cations should exhibit the dielectric loss in the lower MW frequency region, especially below 1 GHz (*24*), and their absorption band of the MW was extended into the high-frequency range as the temperature increased because of the positive activation energy of cation conductivity (*25*).

**Fig. 1.**
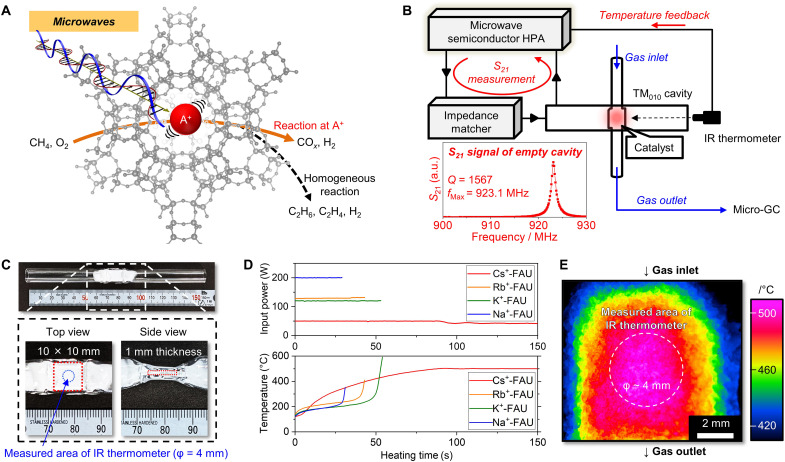
Concepts of MW catalysis. (**A**) Illustration of the selective vibration of alkaline metal cations in a FAU zeolite nanocavity under MW. The vibrated cations catalyze CH_4_ partial oxidation without any gas-phase side reactions. (**B**) Setup of MW irradiation using a TM_010_ cavity (internal diameter = 263 mm, internal height = 20 mm) equipped with an in situ S21 parameter (MW transmission coefficient) measurement system and a temperature feedback system using an IR thermometer. The inset shows the *S*_21_ signal of the empty cavity. (**C**) Photographs of the catalyst bed packed in a quartz tube and partially molded into a square shape (10 mm by 10 mm). The thickness of the catalyst bed was only 1 mm. The IR radiation thermometer monitored the surface temperature of the central part (ϕ = 4 mm) of the packed catalyst. (**D**) Heating profiles of a series of alkali metal cation–incorporated dehydrated FAU-type zeolite under dried N_2_ gas flow (100 ml min^−1^). (**E**) IR thermography camera image of the packed bed of Cs^+^-FAU zeolites stabilized at 500°C.

This paper experimentally demonstrates the selective heating of a single alkaline metal cation incorporated in zeolite pores by L-band MW (~0.9 GHz) to develop an atomic-scale temperature gradient between the cations and the zeolite framework. Among the various zeolite topology, we focused on Faujasite (FAU)–type zeolite because of their large pore window with 12-membered rings, enabling us to introduce various alkaline metal cations by ion exchange method. In addition, FAU-type zeolites are composed of only double six-membered rings (*d*6*r*) as building units, which makes them suitable for x-ray structural analysis due to their good symmetry. If this is prevalent, product selectivity for the CH_4_ combustion would be drastically changed; it should suppress gas-phase C-C coupling reactions in the gas phase of the zeolite micropores, thus markedly increasing the selectivity of carbon oxides by the selectively excited cations (Fig. 1A). A synchrotron-based MW experiment applying in situ high-energy x-ray total scattering (HEXTS) provides direct evidence of peculiar displacements of the selectively heated Cs^+^ cations, consistent with the prediction obtained by an advanced molecular dynamics (MD) simulation that introduces an MW heating simulation.

## RESULTS AND DISCUSSION

A well-defined MW irradiation system (Fig. 1B) was used for the demonstration. A GaN semiconductor MW generator (900 to 930 MHz) equipped with a high-power amplifier was connected to the single transverse magnetic wave mode cavity (TM_010_ mode), where the quality factor (*Q*) was 1567 and the peak top frequency (*f*_max_) was 923.1 MHz (Fig. 1B, inset). A signal sensor for *S* parameter measurement (*S*_21_) mounted on the semiconductor MW generator can simultaneously measure the dielectric properties of the catalysts (*26*–*28*). Typically, a temperature gradient from the core to the surface occurs in a cylindrical shape packed catalyst bed, that is, the core exhibits the highest temperature and then the temperature parabolically decreases toward the surface (*29*–*31*). To avoid the three-dimensional temperature gradient and undetectable hotspots in the depth direction, which prevents accurate temperature measurement (*32*), in this study, the catalyst layer was packed as a flat plate (10 mm by 10 mm) with a thickness of 1 mm (Fig. 1C). The zeolite catalysts were sieved into particles of 500 to 750 μm to avoid multilayer formation with more than two particles in the thickness direction. The pressure gauge installed between the reaction tube and the mass flow controller showed almost atmospheric pressure, suggesting that there was no pressure drop due to the catalyst bed.

The surface temperature of the central part (ϕ = 4 mm) of the packed catalyst was measured with an IR radiation thermometer when heating tests were performed on a series of alkali metal cation (Na^+^, K^+^, Rb^+^, or Cs^+^)–incorporated FAU-type zeolites (Si/Al = 2.8, dehydrated at 500°C) under N_2_ gas flow (Fig. 1D). The IR thermometer was calibrated on the basis of the emissivity of the packed zeolite bed (fig. S1). Among the zeolites investigated, the Cs-incorporated FAU zeolite (Cs^+^-FAU) was most efficiently heated under 50-W MW and stabilized at 500°C under ~40-W MW. Solid-state ^27^Al magic angle spinning (MAS) nuclear magnetic resonance (NMR) showed only a peak assigned to tetrahedral aluminum species substituted in the FAU-type zeolite framework (fig. S2). Moreover, no structural change was observed in scanning electron microscope (SEM) images before and after MW irradiation to heat at 500°C (fig. S3). Thus, irreversible damages on Cs^+^-FAU, e.g., the formation of octahedral extra framework aluminum, did not occur. Two-dimensional temperature distribution of the packed Cs^+^-FAU zeolites kept at 500°C under ~40-W MW was monitored by an IR thermography camera (Fig. 1E). The center part (~4 mm by 6 mm) of the catalyst surface reached 500°C, while the temperature decreased from the center part toward the edges. Hence, the average temperature of the entire packed zeolite was lower than 500°C.

On the other hand, MW power of more than 100 W was needed to heat Na^+^-, K^+^-, and Rb^+^-FAU zeolites above 200°C, and the heating rate increased with temperature rise, suggesting that the dielectric properties (ɛ′ and ɛ″) of these zeolites should increase drastically along with the heating. In comparison, Ca^2+^-FAU was hardly heated even under 250-W MW irradiation (fig. S4, A and B). Thus, divalent cations are less easily heated than monovalent cations, probably because of the inhibition of cation vibration by strong electrostatic force between divalent cation and the zeolite framework. H^+^-FAU did not exhibit temperature rise even under the maximum power (300 W) of the MW generator used. Cs^+^-FAU (Si/Al = 12.5) was heated under MW power (60 W), although it took 170 s to reach 500°C (fig. S4, C and D). The slope was slower than Cs^+^-FAU (Si/Al = 2.8), which reached 500°C in about 80 s under 50-W irradiation. Thus, Cs^+^ density plays an important role in heating efficiency.

The dielectric properties of these zeolites at room temperature and at 500°C under MW were determined from the *S*_21_ signal of the zeolites introduced in the TM_010_ cavity (table S1). However, the dielectric property of Na^+^-FAU was not obtained at 500°C because it was impossible to hold Na^+^-FAU at 500°C because of thermal runaway (*33*), where a rapid increase in the rate of temperature rise, or thermal runaway, occurs at a threshold of 200°C although the electric field remains unchanged. The raw data of the *S*_21_ signal as a function of MW frequency was shown in figs. S5 to S8. In all the cases, the dielectric loss tangent (tanδ) drastically decreased after dehydration because of the loss in the high MW absorption ability of water molecules. Once dehydrated, tanδ was increased at 500°C, and Cs^+^-FAU exhibited the largest dielectric value, followed by Rb^+^-FAU and K^+^-FAU. Therefore, the heating nature depends on the type of cations introduced in the zeolite pores.

**Fig. 2.**
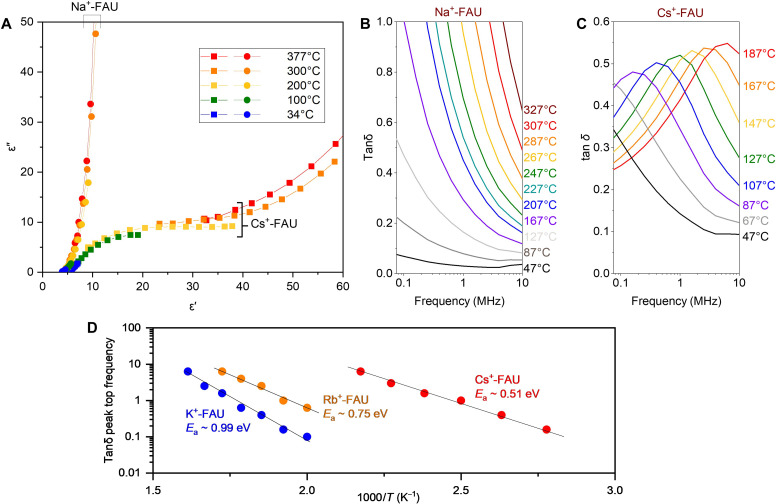
Ion conductivity tests of dehydrated FAU-type zeolite with various alkali metal cations. (**A**) Cole-Cole plots of the Na^+^-FAU and Cs^+^-FAU zeolites measured at various temperatures (frequency range: 0.1 to 10 MHz). (**B** and **C**) Dielectric spectra of the Na^+^-FAU and Cs^+^-FAU zeolite. (**D**) Arrhenius plots of the ion vibration in Cs^+^-, Rb^+^-, and K^+^-FAU zeolites regarding the peak top frequency of tanδ spectra. In all the measurements, zeolite samples were dehydrated at 500°C before measurement.

To discuss the heating mechanism of the zeolites under the MW, dielectric spectra (0.1 to 10 MHz) of the zeolite catalysts were measured at various temperatures using an electric heater under a dried gas condition. Cole-Cole plots of the Na^+^-FAU and Cs^+^-FAU zeolites are shown in Fig. 2A. The feature of the plots of Cs^+^-FAU demonstrated Debye-type dielectric relaxation and the large ɛ″ in the larger ɛ′ region due to the contribution of ionic conduction (*34*). The ɛ″ in the larger ɛ′ region increased with the temperature elevation probably because of activation of ionic conduction. On the other hand, Na^+^-FAU exhibited only the contribution of ionic conduction without any component of dielectric relaxation. Consequently, Na^+^-FAU did not show any peak feature in the spectra of the loss tangent in this frequency region (Fig. 2B), while the spectra of the Cs^+^-FAU showed clear peaks attributed to the dielectric loss from dielectric relaxation (Fig. 2C). Such a conductive property of the Na^+^-FAU might have caused the thermal runaway as shown in Fig. 1D. These differences should be due to the mobile range of ions in the zeolite micropores as discussed later.

Figure 2D shows the peak top frequency of the tanδ as a function of the inverse of measurement temperature in Cs^+^-, Rb^+^-, and K^+^-FAU zeolites. The spectra of the tanδ of Rb^+^- and K^+^-FAU zeolites are shown in fig. S9. From the slope of this plot, the activation energy of the ionic conduction in Cs^+^-FAU can be estimated as 0.51 eV, the value of which is reasonable for the dielectric relaxation originating from ionic vibration in zeolites (*34*). The order of the activation energy of the dielectric relaxation was Cs^+^-FAU < Rb^+^-FAU < K^+^-FAU, which corresponded to the order of the tanδ value of these zeolites at 500°C under 900- to 930-MHz MW. Hence, the MW heating mechanism of the alkali metal cation–incorporated zeolites should be attributed to the dielectric relaxation contributed by ionic conduction, i.e., the alkali metal cation should be the antenna to absorb the irradiated MW energy.

**Fig. 3.**
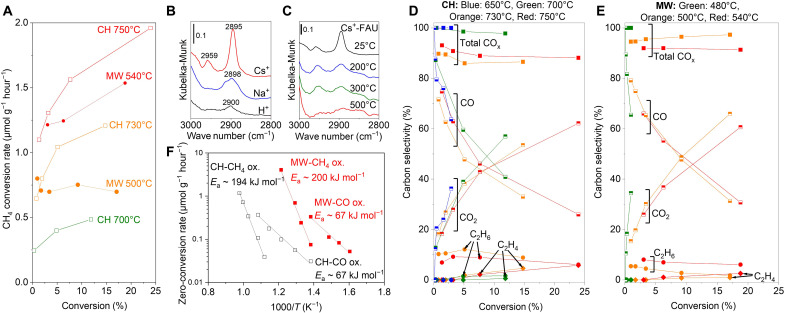
Catalytic activity of Cs^+^-FAU zeolite under CH or MW. (**A**) CH_4_ conversion rate over Cs^+^-FAU as a function of CH_4_ conversion under MW or CH. Total pressure: 101 kPa. Gas composition: 6-kPa CH_4_ and 6-kPa O_2_, N_2_ balance. Total flow rate: 10 to 200 ml min^−1^. Catalyst amount: 100 mg. (**B** and **C**) Results of DRIFTS measurement recorded under 10-kPa CH_4_ flow (total pressure: 101 kPa, Ar balance) after dehydration at 500°C under pure Ar flow. The heating method was CH. (**D** and **E**) Product selectivity of CH_4_ oxidation over Cs^+^-FAU as a function of CH_4_ conversion. (**F**) Arrhenius plots of CH_4_ oxidation or CO oxidation over the Cs^+^-FAU zeolite. Gas composition for CH_4_ oxidation: 6-kPa CH_4_ and 6-kPa O_2_ (total: 101 kPa, N_2_ balance). Gas composition for CO oxidation: 1-kPa CO and 6-kPa O_2_ (total: 101 kPa, N_2_ balance).

Figure 3A shows the CH_4_ conversion rate as a function of CH_4_ conversion over a Cs^+^-FAU zeolite catalyst bed under the CH method using an electric furnace with heat-insulating materials or the MW. The MW rate at the surface temperature of 450° to 550°C detected by an IR thermometer was roughly equivalent to the CH rate at 650° to 750°C monitored by a thermocouple. This should be due to the selective heating of Cs^+^ by MW, which was undetectable by an IR thermometer. While 40 W of MW input was required to maintain the Cs^+^-FAU at 500°C (Fig. 1D), the power required to maintain the temperature at 750°C using a tube electric furnace (see the Supplementary Materials for the detailed information) was 164 W. Thus, MWs could reduce the energy required by a factor of four. The rate in CH drastically increased with increasing CH_4_ conversion, although the furnace temperature was constantly monitored by the thermocouple located close to the catalyst bed. On the other hand, the rate under MW was almost constant as a function of the CH_4_ conversion at the surface temperature of 500°C or slightly increased at 540°C. The kinetic orders of the CH_4_ oxidation with respect to CH_4_ and O_2_ pressures were almost half in CH at 700°C and MW at 500°C (figs. S10 to S15). Therefore, the increase in reaction rate with the conversion should be due to a gas-phase contribution to CH_4_ conversion as discussed later. As a comparison, a CO oxidation reaction over Cs^+^-FAU was demonstrated under CH at 450° to 650°C and MW at 350° to 450°C as shown in figs. S16 and S17, respectively. The CO conversion rate was almost constant under both heating methods because of the absence of the gas-phase contribution on CO conversion at the temperature range. The kinetic orders were almost unity with respect to CO pressure and half with respect to O_2_ pressure in both CH and MW (figs. S18 to S23).

In contrast, Na^+^-FAU and siliceous FAU (Si/Al = 250) showed a much lower CH_4_ conversion performance at 750°C (figs. S24 and S25). Thus, Cs^+^ played a role in CH_4_ activation sites. For further confirmation of the Cs^+^ as an active site for CH_4_ activation, diffuse reflectance IR Fourier transform spectroscopy (DRIFTS) under CH was performed. The DRIFTS result (Fig. 3B) of the Cs^+^-FAU zeolite at room temperature under the 10-kPa CH_4_ flow condition shows two peaks in the C-H vibration region. The strong peak at 2895 cm^−1^ can be attributed to the symmetric *v*_1_ mode of the CH_4_ molecule adsorbed on the Cs^+^ cation (Cs^+^···H-CH_3_) (*35*), while the weak peak at 2959 cm^−1^ can be attributed to the C-H stretching mode of the CH_3_ group adsorbed on Cs cations generated by dissociative adsorption of the CH_4_ molecule (*36*). The *v*_1_ peak was weakened by temperature elevation and disappeared at 500°C even under the 10-kPa CH_4_ flow condition (Fig. 3C). On the other hand, the C-H stretching mode of the CH_3_ group was enhanced at 200°C compared to 25°C, likely because of the activated adsorption of CH_4_ in a C-H bond at elevated temperature, while the peak disappeared at 500°C because of the desorption of the species. The peak of the *v*_1_ mode of the CH_4_ molecule was shifted to a higher wave number in the Na^+^-FAU zeolite, suggesting the weaker interaction between the CH_4_ molecules and Na cations. Moreover, the *v*_1_ peak was splitting into two peaks, which can be attributed to Na^+^ in the different zeolite sites (*35*). A trace amount of the *v*_1_ mode was measured over the H^+^-FAU zeolite, which can be assigned to CH_4_ adsorption on residual Na cations or framework oxygens. These spectroscopic results suggested that the CH_4_ molecules can be activated over Cs cations in the FAU zeolite cavity. Acharyya and coworkers (*37*) found that the Cs^+^ cations confined in the FAU zeolite can activate the C_sp3_-H bonds of CH_4_ and O_2_ molecules to produce oxygen transfer insertion intermediates.

The reaction selectivity under CH or MW was compared as shown in Fig. 3 (D and E). For both heating methods, the CO selectivity extrapolated to zero conversion was approximately 100%, showing that CO was the primary product for the reaction. As the conversion increased, CO selectivity was dropped, and CO_2_ selectivity increased because of the sequential oxidation of CO to CO_2_ over the Cs^+^-FAU zeolite. In the case of CH, the selectivity of the total CO*_x_* decreased with CH_4_ conversion, and C_2_H_4_ and C_2_H_6_ were produced, while the selectivity of CO*_x_* under MW was maintained at 540°C or even increased close to ~100% at 500°C as a function of CH_4_ conversion. The formation mechanism of C_2_H_6_ should be reactions of the coupling reaction of methyl radicals (CH_3_·) in the gas phase as follows (*38*)

$$2{\mathrm{CH}}_{3}\cdot \to C_{2}H_{6}$$

To discuss the gas-phase contribution for CH_3_· generation, the reaction kinetics of the CH_4_/O_2_ mixture (6 kPa each, N_2_ balanced) in a homogeneous plug-flow reactor without any catalyst was simulated using a state-of-the-art gas-phase chemical kinetic mechanism (*39*). Figure S26A shows that the rates of four major pathways for CH_3_· generation from CH_4_ increased with CH_4_ conversion at 750°C. Because zero-extrapolated C_2_H_6_ selectivity was ~100% (fig. S26B), the primary product for the gas-phase radical propagation of CH_4_ should be C_2_H_6_. The CO and C_2_H_4_ selectivity in the gas-phase reaction increased with the CH_4_ conversion, suggesting that these products were generated by the sequential dehydrogenation reaction of C_2_H_6_. The drastic increase in the reaction rate with high CH_4_ conversion under CH should be induced by the gas-phase radical propagation to convert CH_4_. When the catalyst surface temperature was maintained at 500°C under MW, the rate was constant as a function of CH_4_ conversion because of the suppression of the gas-phase contribution, suggesting that MW localize their energy onto Cs^+^ to induce the heterogeneous reaction. Consequently, the C_2_ formation under MW was suppressed compared to that under CH. The slight contribution of radical propagation in the gas phase was observed when the catalyst surface was maintained at 540°C under MW.

Figure 3F shows the Arrhenius plots of the CH_4_ oxidation and CO oxidation reaction using the zero-conversion extrapolated rate, where the rate extrapolated to zero conversion was used to cancel out the gas-phase contribution. The temperature under MW irradiation is the surface temperature measured with an IR radiation thermometer as shown in Fig. 1E. The apparent activation energy (*E*_a_) was also not changed by the MW. The photon energy of MWs is less than 1 meV, making it physicochemically impossible for them to directly affect the kinetics of chemical reactions. Although the reaction pathways and mechanisms were not affected by the MW, which corresponds to the previous reports (*40*, *41*), the reaction rate increased because of the MW selective heating of single Cs^+^ in the zeolite cavity. Therefore, the rate enhancement under MWs indicated the local hotspots at catalytic active sites, which are undetectable using the IR radiation thermometer. From the Arrhenius plot, the temperature at Cs^+^ was estimated to be about 150° to 250°C hotter than the surroundings under MW.

To confirm the selective heating of a single Cs cation in the FAU zeolite cavity, in situ x-ray measurements under MW were performed at the SPring-8 synchrotron facility (Hyogo, Japan). While several in situ x-ray analytical methods under MW have been reported (*42*), we focused on HEXTS measurement coupled with pair distribution function (PDF) analysis (*43*) and x-ray absorption spectroscopy (XAS) measurements. A pelletized Cs^+^-FAU disk with a thickness of 0.75 mm fixed in the quartz tube was used as the specimen for the synchrotron measurements (fig. S27). On the basis of a previous report (*17*), the MW-induced temperature difference between the center and the surface in the thickness direction for the 0.75-mm thick disk can be about 5°C at most. On the other hand, decreasing temperature from the center to the sides in the in-plane direction of the disk can be assumed under the MW, which might result in a temperature difference of several tens of degrees. To avoid such temperature inaccuracies, the temperature at the center of the circular surface of the disk-shaped sample was measured by the IR thermometer placed downstream of the x-ray beam. The spot for temperature measurement was a ϕ = 4 mm diameter circle, which is much larger than the x-ray beam spot (2 mm–by–2 mm square for HEXTS and 0.5 mm–by–3 mm rectangular for XAS). For the detailed experimental setup, see Materials and Methods and figs. S28 to S31 for HEXTS and fig. S35 for XAS.

**Fig. 4.**
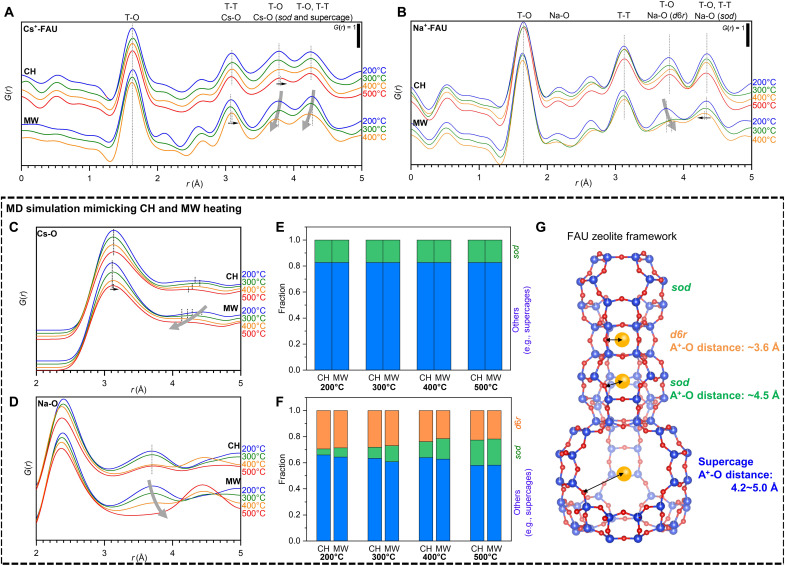
MW in situ synchrotron characterizations of selective heating of Cs^+^ cations. (**A** and **B**) In situ HEXTS results of Cs^+^-FAU and Na^+^-FAU zeolites under CH and MW. (**C** and **D**) Estimated PDF of Cs-O in the Cs^+^-FAU zeolite and Na-O in Na^+^-FAU under CH and MW by MD calculation. Other PDF results were shown in figs. S34 and S35. (**E** and **F**) Fraction of the Cs^+^ and Na^+^ location in the zeolites within 1-ns MD simulation. (**G**) FAU zeolite framework structure. Blue, red, and orange balls represent the T atoms (Si or Al), O, and alkaline cations, respectively.

Figure 4 (A and B) shows the reduced PDF, *G*(*r*), of Cs^+^-FAU and Na^+^-FAU under CH and MW, respectively. These *G*(*r*) values were obtained by implementing Fourier transformation of the total scattering factor, *S*(*q*), as shown in figs. S32 and S33. See Materials and Methods for the detailed calculation procedure. Note that T in Fig. 4 (A and B) denotes the tetrahedrally coordinated silicon or aluminum atoms in the zeolite framework. In the case of Cs^+^-FAU, characteristic shifts under MW were observed in the peaks attributed to the PDF around Cs^+^, that is, the peak at 3.1 Å attributed to the T-T or Cs-O pair was shifted to longer, while the T-O or Cs-T pair peak at 3.8 Å and the T-O, T-T, or Cs-O pair peak at 4.3 Å was shifted to shorter as the temperature rose under MW. On the other hand, different characteristic peak shifts under MW were detected in Na^+^-FAU, where the peak at 3.8 Å attributed to the Na-O pair showed a long-distance shift and decrease in peak intensity with increasing temperature under MW, while the peak hardly shifted under CH. These characteristic peak shifts should not be caused by inaccurate temperature measurement due to macroscopic hotspots in the specimen. Rather, they should be induced by essential atomistic local structural changes caused by the selective heating of Cs^+^ and Na^+^ by MW.

For a detailed interpretation of the in situ HEXTS results, MD simulations of Cs^+^-FAU and Na^+^-FAU zeolites were performed. To mimic the local heating of Cs^+^ and Na^+^ in the FAU zeolite cavity under MW, Cs and Na atoms were heated to 350°, 475°, 630°, and 750°C, while the average temperature of the whole structure was kept at 200°, 300°, 400°, and 500°C, respectively, estimated from the Arrhenius plots shown in Fig. 3E. Simulations at these different temperatures correspond to experimentally varying the intensity of the incoming MWs. The simulated PDF of the Cs-O pair (Fig. 4C) reproduced the characteristic shift under MW, that is, the long-distance shift of the peak at 3.1 Å and the short-distance shift of the broad peak around 4.3 Å. In contrast, the simulated PDF shown in the Na-O pair (Fig. 4D) well reproduced the characteristic long-distance shift and intensity decrease of the peak 3.8 Å under MW. The excellent agreement of the peak shift trends between MD calculations and the HEXTS experiments is strong evidence that the Cs^+^ and Na^+^ were selectively heated by MW. Figure 4 (E and F) shows the fraction of the Cs^+^ and Na^+^ location in the FAU zeolite within a 1-ns MD simulation, respectively.

The MD simulation movies of Cs^+^- and Na^+^-FAU zeolites under CH or MW within 200 ps were provided as movies S1 to S4. Cs^+^ did not enter the double six-membered rings (*d6r*; Fig. 4G), resulting in the fraction of Cs^+^ in the sodalite cage (*sod*) and supercage being independent of the heating method and temperature. On the other hand, Na^+^ was tapped out of *d*6*r* and moved to *sod* and the supercage at a higher temperature and tended to be more easily expelled from *d*6*r* by MW. Therefore, the peak shift around 4.3 Å occurs because Cs^+^ is likely to be rampant in the supercage and *sod*, while the peak at 3.8 Å in Na^+^-FAU was reduced because the Na^+^ in *d*6*r* is transferred to the supercage and *sod*. These differences in the behavior of the alkali cations explain the difference in dielectric properties as shown in Fig. 2, suggesting that designing catalysts with a combination of large cations (e.g., Cs^+^) and host frameworks (e.g., FAU zeolite) to confine cations into specific cages enables us to efficiently induce selective heating of a single cation.

In summary, we demonstrated the selective heating of alkali cations incorporated in an aluminosilicate FAU zeolite cavity under 915-MHz MW using the in situ complex dielectric parameter evaluation technique and in situ synchrotron characterization method. Among the alkali cation–exchanged FAU zeolites, Cs^+^-exchanged zeolite was efficiently and stably heated by MW because of the large dielectric constant at the largest atomic mass. The MW selective heating of Cs^+^ achieved a selectivity improvement in the catalytic CH_4_ combustion reaction at Cs^+^ sites by suppressing gas-phase CH_4_ radical propagation. This study provides experimental evidence of local heating effects occurring at the atomic level, beyond the nanoscale MW local heating effects that have been discussed in composite material systems. Furthermore, the clarification of the effect of MW on catalytic reactions at the atomic scale opens the science of “microwave catalysis” (*44*). Understanding the local heating field on the atomic scale will provide a direction for a precise mechanistic understanding of the ambiguous term microwave “nonthermal” effects. These findings are expected to lead to energy-saving catalytic systems that can be realized by concentrating diffusive thermal energy locally and to advanced materials chemistry that requires atomic-scale control of states.

## MATERIALS AND METHODS

### Preparation and characterization of alkali metal cation–exchanged FAU zeolites

The starting Na^+^-type FAU zeolite (HSZ-320NAA; Si/Al = 2.8; mean particle size, ~300 nm; specific surface area, ~660 m^2^ g^−1^) was purchased from Tosoh Corporation and used as supplied. Alkali metal cation–exchanged FAU zeolites were prepared by the conventional liquid-phase ion exchange method. Cesium nitrate (CsNO_3_; 99.9%; FUJIFILM Wako Pure Chemical Corporation), rubidium nitrate (RbNO_3_; 98.0+%; FUJIFILM Wako Pure Chemical Corporation), or potassium nitrate (KNO_3_; 99.0+%; FUJIFILM Wako Pure Chemical Corporation) was dissolved in ultrapure water (0.2 M and 30 ml). The FAU zeolite (100 mg) was dispersed in the prepared alkali nitrate aqueous solution and then shaken at 80°C for 2 hours. The dispersed zeolite was recovered using centrifugation and redispersed in fresh alkali nitrate solution (0.2 M each and 30 ml). After this procedure was repeated three times, the zeolite was washed with a large amount of ultrapure water and then calcined at 550°C under static air overnight at a ramp rate of ~10°C min^−1^. Consequently, the Cs^+^-, Rb^+^-, or K^+^-FAU zeolite was obtained. The extent of the ion exchange was estimated by inductively coupled plasma optical emission spectroscopy using iCAP 300 (Thermo Fisher Scientific Inc.) and flame atomic absorption spectroscopy using Z-2000 (Hitachi High-Tech Corporation). The elemental composition of zeolites was summarized in table S2. SEM images were recorded using JSM-IT800 (JEOL Ltd.). Solid-state MAS NMR measurements were taken by a JNM-ECA 500 instrument (JEOL Ltd.). The ^27^Al MAS NMR spectra were recorded at 130.33 MHz with a π/2 pulse length of 3.2 μs, a recycle delay of 5 s, and a spinning frequency of 14 kHz.

### MW irradiation setup

The illustration of the MW irradiation setup was shown in Fig. 1A. An L-band GaN semiconductor MW generator equipped with a high-power amplifier (Ryowa Electronics Co. Ltd.) was connected to the single transverse magnetic wave mode cavity (TM_010_ mode, cylindrical) through an impedance-matching device. The surface temperature of the catalyst bed was monitored using an IR radiation thermometer (FLHX series; Japan Sensor Corporation). The measured wavelength was 0.8 to 2.6 μm, where the transmittance of the quartz tube was almost unity (>90%). Two-dimensional temperature distribution of the catalytic bed was recorded using the IR thermography camera, InfReC H9000 Thermo HAWK (Nippon Avionics Co. Ltd., Tokyo, Japan).

A signal sensor for *S* parameter measurement (*S*_21_) was mounted on the semiconductor MW generator. The dielectric constant (ɛ′), dielectric loss factor (ɛ″), and dielectric loss tangent (tanδ) were calculated by the following equations

$${\varepsilon_{r}}^{\prime }=1+\frac{1}{\alpha_{n}}\frac{f_{0}-f_{L}}{f_{0}}\frac{V}{\text{Δ}V}$$ (1)

$${\varepsilon_{r}}^{\prime \prime }=\frac{1}{\alpha_{n}}\left(\frac{1}{Q_{L}}-\frac{1}{Q_{0}}\right)\frac{V}{\text{Δ}V}$$ (2)

$$Q_{L\;\mathrm{or}\;0}=\frac{f_{L\;\mathrm{or}\;0}}{\text{Δ}f_{L\;\mathrm{or}\;0}}$$ (3)

$$\tan\;\delta =\frac{{\varepsilon_{r}}^{\prime \prime }}{{\varepsilon_{r}}^{\prime }}$$ (4)

where the subscripts “*L*” and “0” represent the parameter before and after insertion of the sample, respectively. *Q*, *f*, and Δ*f* denote the quality factor, the peak top frequency, and the full width at half maximum of the *S*_21_ parameter, respectively. α*_n_* is the constant determined by a resonance mode and fixed as 1.85515 for TM_010_ mode in this manuscript. *V* represents the internal volume of the TM_010_ MW cavity, and Δ*V* is for the sample volume.

### Dielectric spectroscopy measurement

The dielectric measurements were performed with a Keysight 4284A and 4285A Precision LCR meter as a function of temperature. The synthesized alkali metal cation–exchanged zeolites (50 mg) were pelleted into 7-mm-diameter disks under 20 MPa and used as specimens. Silver-thin layers were deposited on both sides of the disks and used as electrodes for high-frequency application. The temperature of the samples was controlled using LINKAM THMS600 for the temperature ranges over 760 to 300 K under dried inert gas. The dielectric constant and dielectric loss factor were measured while the temperature was raised and cooled repeatedly in an inert gas atmosphere. We confirmed that the change in dielectric constants can be measured reproducibly by sufficiently desorbing adsorbed water and other substances through repeated heating and cooling. After that, the dielectric constants were recorded for use in the manuscript.

### Catalytic performance test

Catalytic performance tests were carried out under atmospheric pressure using a plug-flow reactor. Before the reaction, the zeolite samples were molded into particles of 500 to 850 μm. Under the CH condition, the temperature of the catalyst bed was measured using a K-type thermocouple inserted into the electric heater (ARF1-200-40KC, ASH Co. Ltd.; inside volume, 15 cm long and a diameter of 8 cm; insulated with a 10-cm-thick insulation material). For the tests under MW, the surface temperature of the catalyst bed was measured by an IR thermometer (FLHX series; Japan Sensor Corporation). Before the catalytic performance test, the catalyst bed was pretreated at 500°C under dry N_2_ flow (99.9999% and 100 ml min^−1^) for dehydration of zeolites. The gas composition for the CH_4_ oxidation test was 2- to 12-kPa CH_4_ (99.9999%) and 2- to 10-kPa O_2_ (99.9999%) balanced with N_2_. For the CO oxidation test, the reactant gas composition was 0.3- to 1.4-kPa CO (99.9999%) and 4- to 10-kPa O_2_ (99.9999%) balanced with N_2_. The contact time of the reactant gas with the catalyst bed was controlled by varying the flow rate in the range of 10 to 200 ml min^−1^. The outlet gas composition was quantitatively analyzed by online Micro GC FUSION (INFICON Co. Ltd) equipped with a thermal conductivity detector.

### Gas-phase reaction simulation

Simulations were performed in CHEMKIN (ANSYS CHEMKIN-PRO v. 19.1, 2020.) using the isothermal plug-flow reactor module. Simulations were conducted at 101-kPa pressure (6-kPa CH_4_ and 6-kPa O_2_, N_2_ balanced) and 750°C with varying residence times. The gas-phase chemical kinetic model used here is the (KAUST-Aramco PAH Mech 1-GS, KAM1-GS). The following paragraphs describe the kinetic model and boundary conditions in detail. The chemical kinetic model KAM1-GS used here was developed using a hierarchical approach (*45*) and consists of 574 species and 3379 reactions. The CH_4_ conversion and selectivity of carbon-containing products are based on a carbon basis as cumulative integral values.

### Diffuse reflectance IR Fourier transform spectroscopy

DRIFTS measurements were carried out using a Fourier transform IR spectrophotometer, FT/IR-6600 (JASCO Corporation), with a liquid nitrogen–cooled mercury cadmium telluride detector. The gas flow and heating for the catalyst powder sample were performed using a heat-vacuum diffuse reflection cell, Heat Chamber Type–1000°C (S.T. Japan Inc.). Pelletized powder samples were placed at the focal point of an integrating sphere. Before the measurement, the powder sample was dehydrated at 500°C for 1 hour under the Ar flow condition (99.9999% and 100 ml min^−1^). Then, the samples were cooled to room temperature, and baseline collection was performed. Subsequently, 10 volume % CH_4_ balanced by Ar (100 ml min^−1^) was introduced into the cell for 1 hour, and the spectrum was recorded. After that, the sample was heated to 200°, 300°, and 500°C under 10 volume % CH_4_ flow. Following this, spectra were recorded at each temperature.

### In situ HEXTS measurement

HEXTS measurements were taken at the BL04B2 beamline high-energy x-ray scattering beamline at the SPring-8 synchrotron facility (Hyogo, Japan). The storage ring was operated at 8 GeV with a ring current of ~99.5 mA in top-up mode. An incident photon energy was monochromated using Si(220) as 61.117 keV (λ = 0.2029 Å). The x-ray scattering was recorded using CdTe and Ge detectors mounted on a diffractometer. The incident x-ray intensity (*I*_0_) was monitored by the ionization chambers filled with 100% Ar. The sample was formed into a thin disk (thickness, ~0.75 mm; fig. S27) and then fixed in a quartz tube with an inner and outer diameter of 7 and 9 mm, respectively. The quartz tube was inserted into the larger-diameter quartz tube with an inner and outer diameter of 10 and 12 mm, respectively. Thus, the disk-shaped sample was fixed in a double-tube structure. By introducing Ar gas (100 ml min^−1^) into the outer large quartz tube, the sample was kept in an Ar atmosphere.

The photograph for in situ HETXS measurement under the conventional heat transfer method was shown in fig. S28. A homemade aluminum heating block [60 mm (length) by 100 mm (width) by 30 mm (height); illustrated in fig. S29] was used to heat the sample. The heating block had two holes, 12 mm in diameter, into one of which was inserted a quartz tube loaded with the sample connected to an Ar gas line (100 ml min^−1^). Into the other, a cartridge heater was inserted. The surface of the heating block was insulated by calcium silicate plates with a thickness of 25.4 mm. The sample temperature was monitored using a K-type thermocouple inserted in the heating block. Simultaneously, the surface temperature of the disk sample was monitored using the IR thermometer, which was used for MW irradiation experiments. To prevent noise in the x-ray scattering patterns, no solid or liquid materials, including quartz tubes, were allowed to exist downstream from the sample. Using this heating block, maximum 2θ was achieved as 45°, which corresponds to a *q*_max_ of 23 Å^−1^ (*q* = 4πsinθ/λ).

To collect the scattering patterns of the samples under MW, the TM_010_ MW cavity connected to a 900- to 930-MHz MW generator was placed on a stage of two-axis diffractometer as shown in fig. S30. As illustrated in fig. S31, the quartz tube with a pelletized sample was placed at the center of the MW cavity and connected to an Ar gas line (100 ml min^−1^). The scattered x-ray beam can be taken out through a 30-mm-diameter hole in the MW cavity. Using this optical system, maximum 2θ was achieved as 43°, which corresponds to a *q*_max_ of 22 Å^−1^.

To confirm that the sample is not experiencing water adsorption due to air inflow from the open side, it is known that the peak intensity of the low-angle x-ray diffraction peaks of zeolites is known to increase with dehydration because the electron scattering density in the zeolite channel is reduced by the desorption of water molecules (*46*). As shown in figs. S32 and S33 (enlarged, the diffraction peaks of Cs^+^-FAU and Na^+^-FAU in the range of 0.3 to 1.0 Å^−1^), the peak intensity was almost unchanged during the measurement, suggesting that the no hygroscopic absorption of zeolite by air pollution has occurred.

The obtained scattering patterns were corrected and normalized by various factors to calculate the total structure factor, *S*(*q*) (*47*, *48*). The reduced PDFs, *G*(*r*), were obtained by implementing Fourier transformation, as expressed in the following equations

$$G(r)=\frac{2}{\pi }\int_{q\min}^{q\max}q[S(q)-1]M(q)\sin(qr)dq$$ (5)

$$M(q)=\frac{\sin(q\text{Δ}r)}{q\text{Δ}r}$$ (6)

where *q*_max_ was set as 20 Å^−1^ for all the experiments.

### In situ x-ray absorption fine structure spectroscopy

Cs K-edge (~36 keV) x-ray absorption fine structure (XAFS) measurement was also performed at the SPring-8 synchrotron facility (Hyogo, Japan) using a BL01B1 beamline. The incident x-ray was monochromated using Si(311) double crystal, and the energy was calibrated using a Cs_2_CO_3_ powder for an edge energy of 35.989 keV. Nondiluted Cs^+^-FAU (25 mg) was pelletized in quartz tubes with an inner diameter of 7 mm and formed into a thin disk (thickness, ~0.75 mm; fig. S26). The beam cross section at the sample position was 0.1 mm by 3 mm. Photographs of the setup for the in situ x-ray absorption measurement were shown in fig. S35. For in situ measurement under a conventional conductive heating cell, the sample was placed in a quartz cell with polyimide windows connected to an N_2_ gas cylinder (100 ml min^−1^). The sample temperature was monitored using a K-type thermocouple inserted in the cell. For in situ measurement under MW, the cylindrical TM_010_ MW cavity connected to a 900- to 930-MHz MW generator was placed perpendicular to the x-ray optical path. The sample was placed in the center of the TM_010_ cavity, which is on the x-ray optical path, and kept under the N_2_ gas flow condition (100 ml min^−1^). The surface temperature of the disk pellet was monitored by the IR thermometer placed downstream of the x-ray beam (see fig. S35B).

The x-ray absorption spectra were recorded via transmittance mode using ionization chambers. The ionization chambers were filled with 75% Ar and 25% Kr mixed gas and 100% Kr to monitor the incident x-ray (*I*_0_) and transmitted x-ray (*I*_1_), respectively. The baseline correction of the x-ray absorption results and extraction and Fourier transformation of EXAFS spectra were performed using the Athena interface for the Demeter software package. The *k*^2^-weighted EXAFS spectra were used for obtaining Fourier transformed EXAFS (FT-EXAFS) spectra with a *k* range of 3 to 8 Å^−1^. The frequency cutoff (*R*_bkg_) was set to 1.7 Å, with an assumption that the nearest FT-EXAFS peak should appear at >1.7 Å based on the previous literature (*49*, *50*).

### MD calculation

The crystal structure of aluminosilicate FAU zeolite was derived from a previous study (*51*): The location of Al atoms best fits with ^29^Si MAS NMR of currently used HSZ-320NAA; the location of Na was optimized by a Monte Carlo simulation using SLC potential (*51*). Na atoms of the structure were replaced with Cs atoms to create the initial structure of the MD simulations of Cs^+^-FAU. That structure underwent MD runs for 1000 ps with a time step of 5 fs with PreFerred Potential version 3.0.0 with D3 correction. To mimic MW heating, we heated Cs atoms to 350°, 475°, 630°, and 750°C, keeping the temperature of the whole structure under 200°, 300°, 400°, and 500°C, respectively, with the NVT ensemble as implemented in the LAMMPS package. Equilibrium MD runs with the NVT ensemble under 200°, 300°, 400°, and 500°C were also performed for comparison. Radial distribution functions were calculated using vasppy software version 0.6.3.0 (*52*).
