# Supplementary material for: Direct microwave energy input on a single cation for outstanding selective catalysis
Source: Sci Adv. 2023 Aug 18;9(33):eadi1744. doi: 10.1126/sciadv.adi1744 (PMC10438448; doi:10.1126/sciadv.adi1744)
Supplement: Supplementary file 1 — Materials and methods Figs. S1 to S40 Tables S1 and S2 Legends for movies S1 to S4 [file sciadv.adi1744_sm.pdf]

Science Advances
AAAS

Supplementary Materials for

Direct microwave energy input on a single cation for outstanding selective catalysis

Fuminao Kishimoto *et al.*

Corresponding author: Fuminao Kishimoto, kfuminao@chemsys.t.u–tokyo.ac.jp;
Kazuhiro Takanabe, takanabe@chemsys.t.u–tokyo.ac.jp

Sci. Adv. **9**, eadi1744 (2023)
DOI: 10.1126/sciadv.adi1744

The PDF file includes:

Materials and methods
Figs. S1 to S40
Tables S1 and S2
Legends for movies S1 to S4

Other Supplementary Material for this manuscript includes the following:

Movies S1 to S4

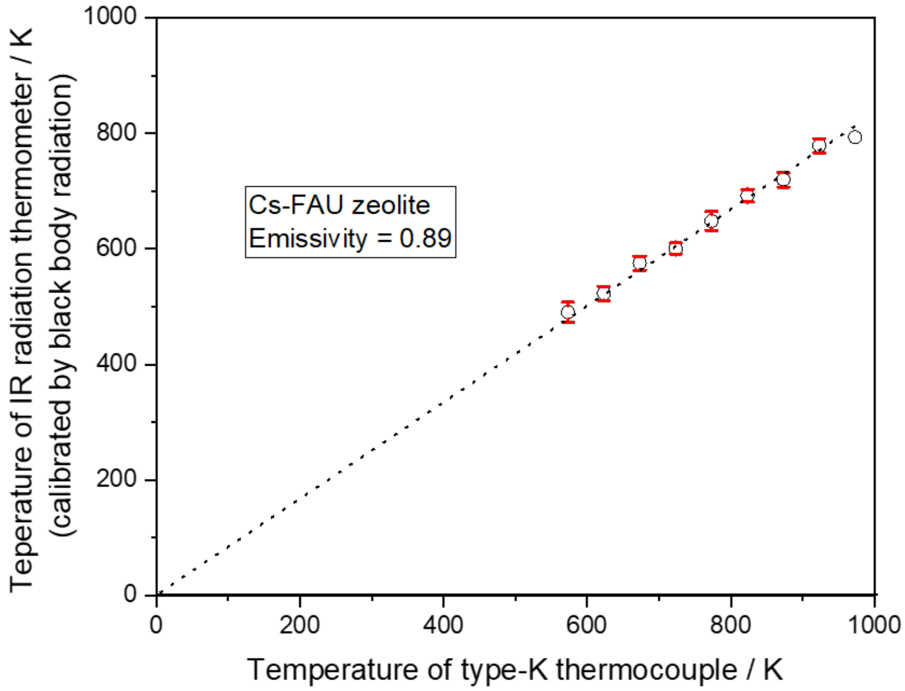

Fig. S1| Emissivity calibration results for IR thermometer. **A** Cs^+-FAU granule (500–750 μm) was packed in a cylindrical stainless-steel tube (internal diameter = 8 mm) and heated by an electric furnace. The IR stray emission from the electric heater equipped inside the furnace was blocked by the stainless-steel body. The temperature of the catalyst bed was measured by a type-K thermometer inserted in the catalyst bed. Then the surface temperature of the catalyst bed was monitored by an IR thermometer calibrated by the standard black body radiation (emissivity = 0.94; JSC-3, Japan Sensor Corporation). The plotted values and error bars are the average and standard deviation of the three measurements.

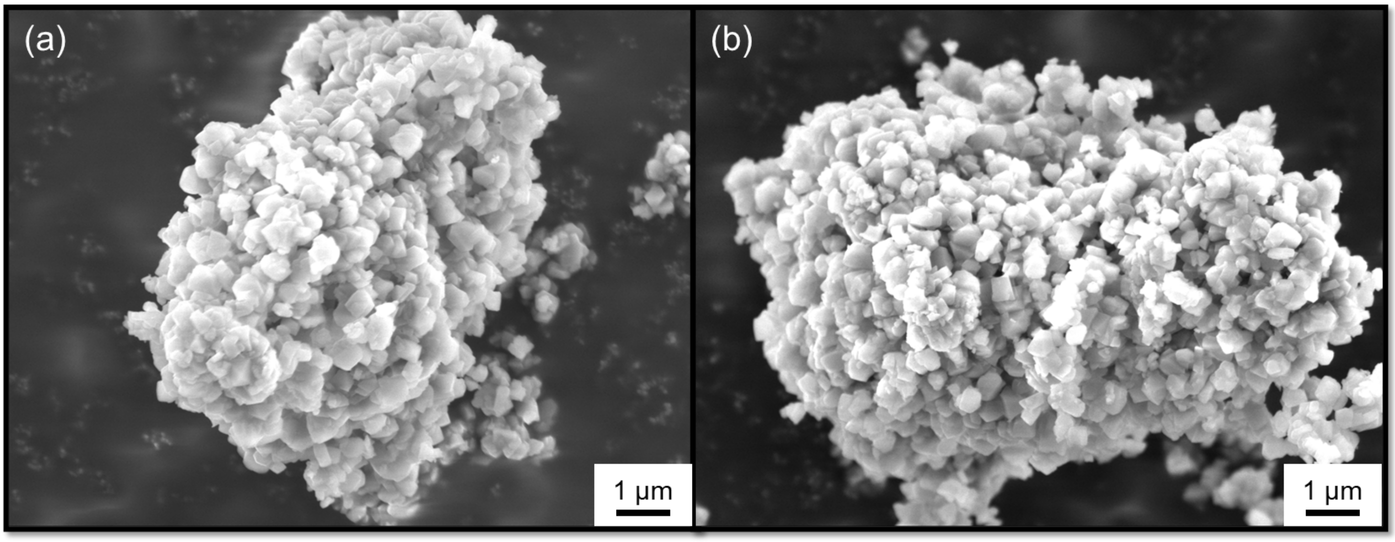

Fig. S2| SEM images of Cs^{+}-FAU zeolite (a) before and (b) after MW irradiation for heating at 500 °C.

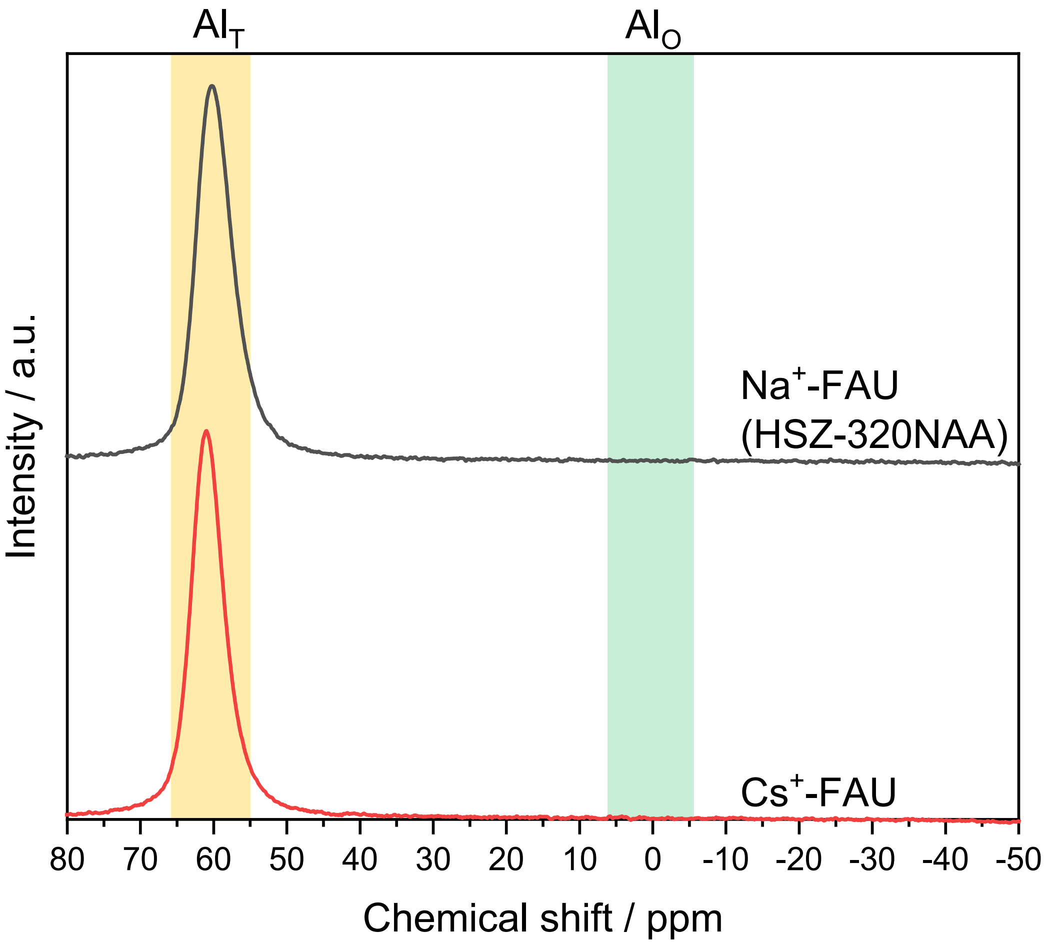

Fig. S3| ^{27}Al MAS NMR spectra of original HSZ–300NAA (Na$^+$-FAU) and Cs exchanged HSZ–320NAA (Cs$^+$-FAU). Al$_T$ and Al$_O$ denote the peaks of aluminum at tetrahedral and octahedral coordination, respectively.

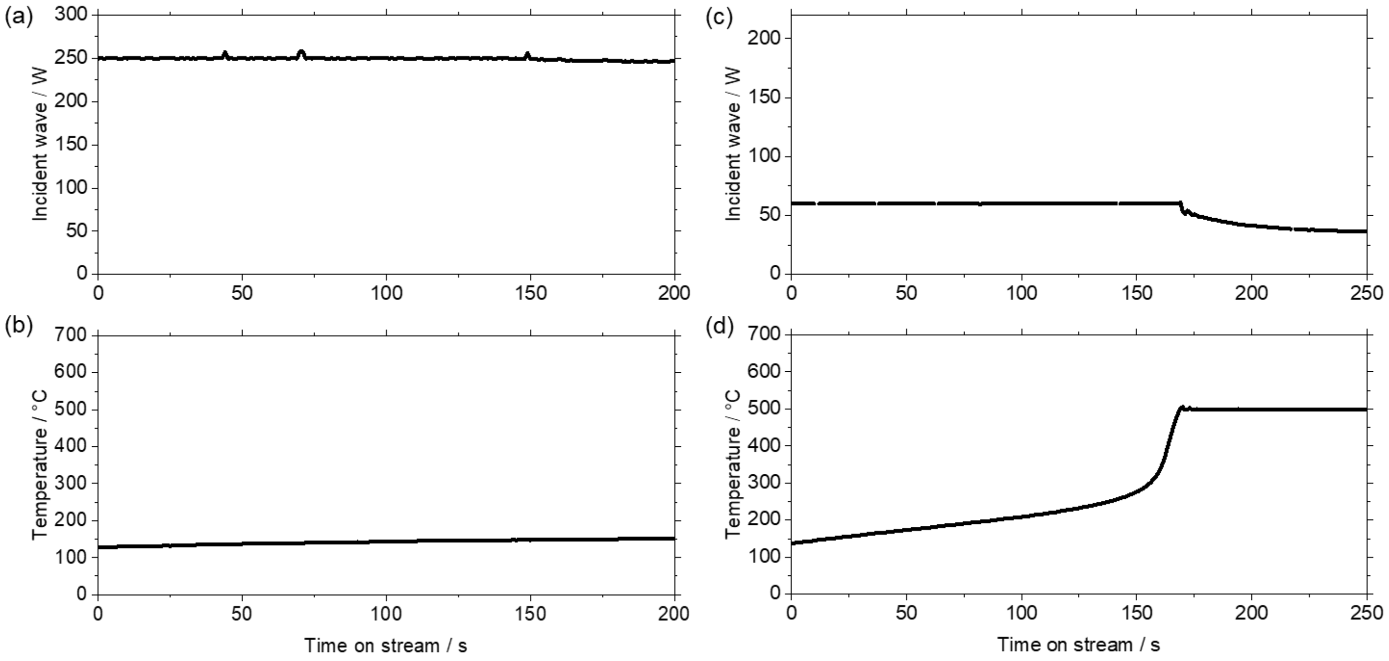

Fig. S4| Microwave heating property of Ca^+-FAU (Si/Al = 2.8) and Cs^+-FAU (Si/Al = 12.5). (a, c) Incident microwave power profile and (b, d) corresponding heating profile of Ca^+-FAU (Si/Al = 2.8) and Cs^+-FAU (Si/Al = 12.5), respectively, under dried N_2 gas flow (100 mL min^{-1}).

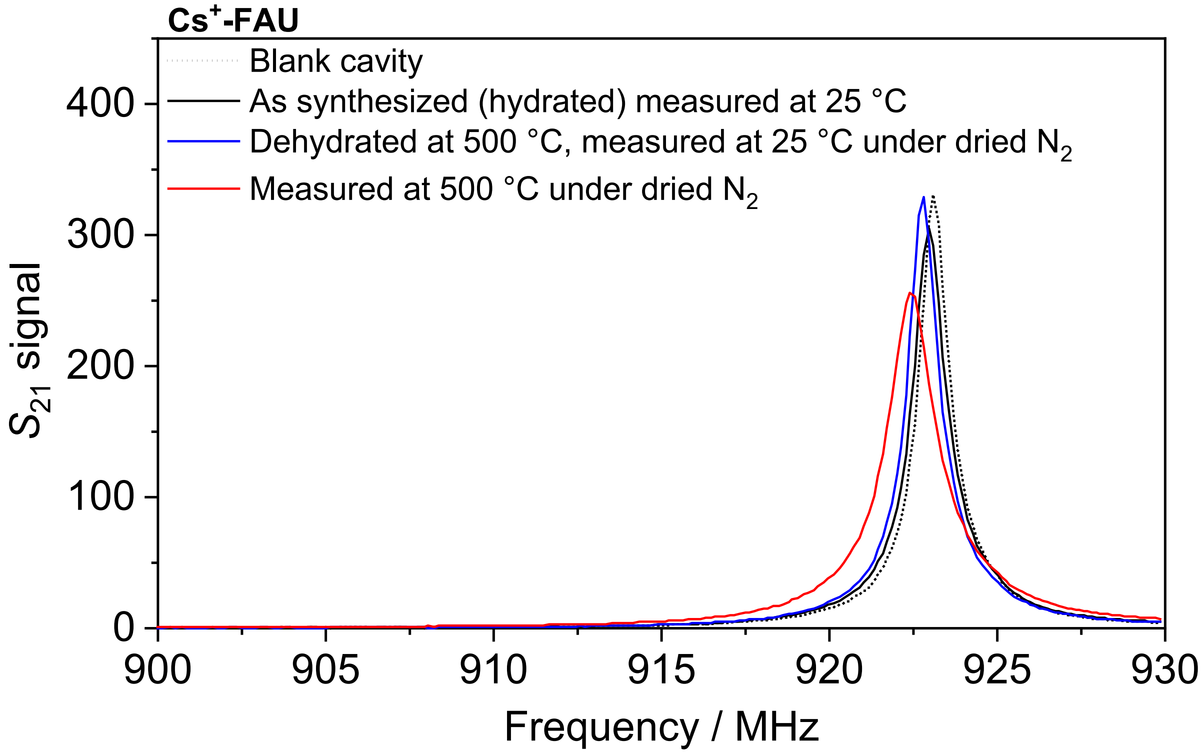

Fig. S5| S_{21} parameter of Cs^{+}-FAU zeolite inserted in TM_{010} cavity. Blank cavity: Q factor = 1567.21, f_{max} = 923.09 MHz; As synthesized Cs^{+}-FAU at 25 °C (100 mg): Q factor = 1396.32, f_{max} = 922.97 MHz; Dehydrated Cs^{+}-FAU at 25 °C under dried N_2 flow (100 mg): Q factor = 1483.62, f_{max} = 922.81 MHz; Cs^{+}-FAU at 500 °C under dried N_2 flow (100 mg): Q factor = 947.99, f_{max} = 922.39 MHz. The calculated real and imaginary parts of the permittivity (ε' and ε'', respectively) and the dielectric loss factor (tanδ) were summarized in Table S1.

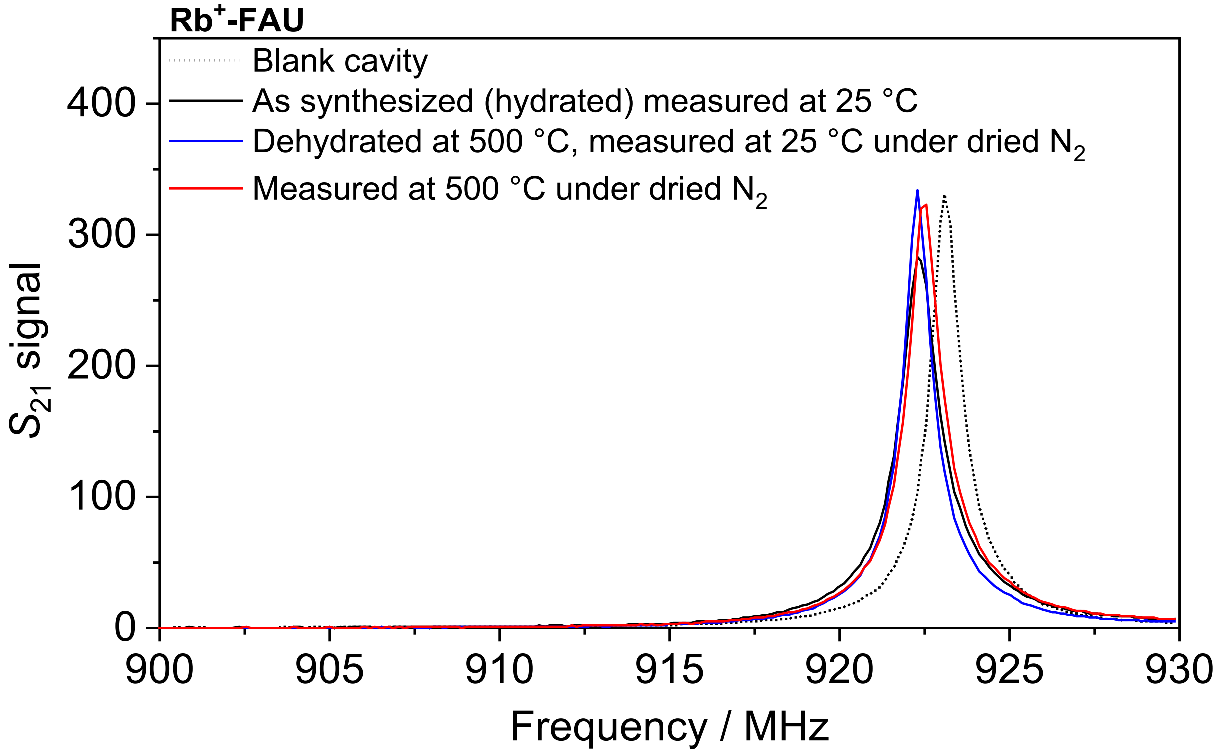

Fig. S6| S_{21} parameter of Rb^{+}-FAU zeolite inserted in TM_{010} cavity. Blank cavity: Q factor = 1567.21, f_{max} = 923.09 MHz; As synthesized Rb^{+}-FAU at 25 °C (100 mg): Q factor = 1191.59, f_{max} = 922.29 MHz; Dehydrated Rb^{+}-FAU at 25 °C under dried N_2 flow (100 mg): Q factor = 1565.85, f_{max} = 922.29 MHz; Rb^{+}-FAU at 500 °C under dried N_2 flow (100 mg): Q factor = 1342.87, f_{max} = 922.55 MHz. The calculated real and imaginary parts of the permittivity (ε' and ε'', respectively) and the dielectric loss factor (tanδ) were summarized in Table S1.

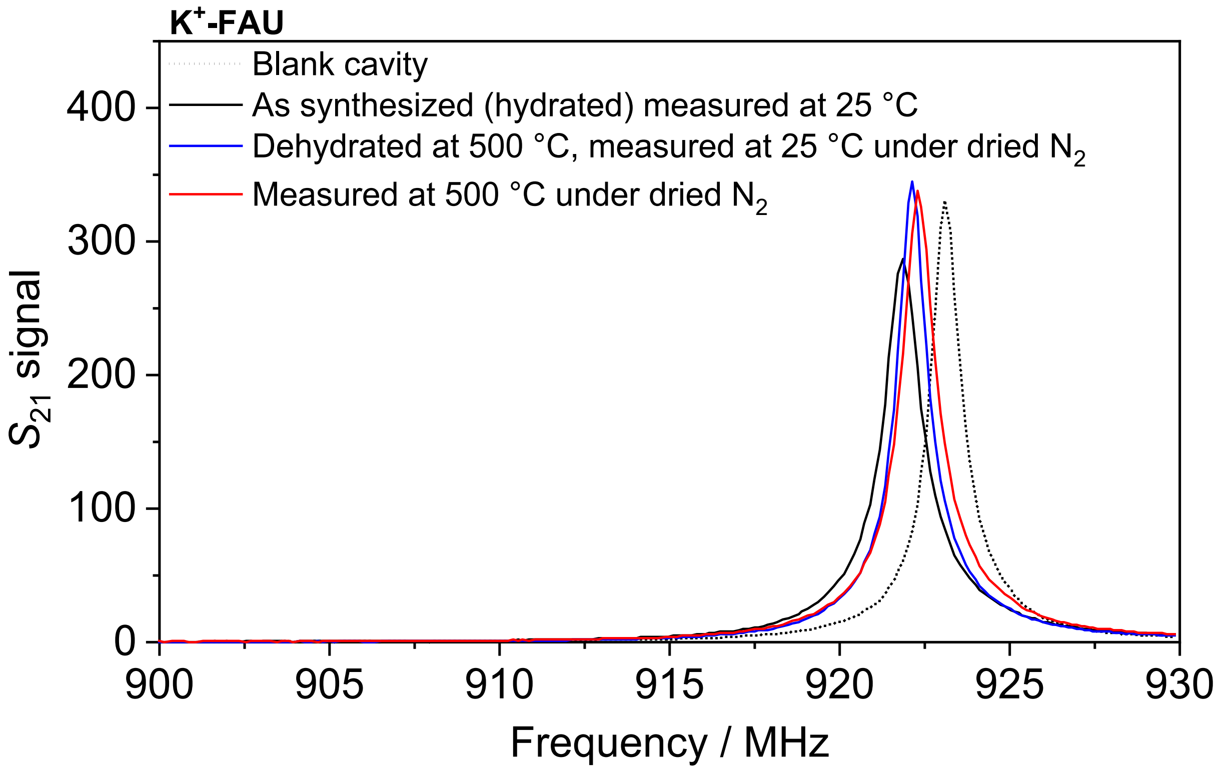

Fig. S7| S_{21} parameter of K^+-FAU zeolite inserted in TM_{010} cavity. Blank cavity: Q factor = 1567.21, f_{max} = 923.09 MHz; As synthesized K^+-FAU at 25 °C (100 mg): Q factor = 1209.79, f_{max} = 921.86 MHz; Dehydrated K^+-FAU at 25 °C under dried N_2 flow (100 mg): Q factor = 1516.66, f_{max} = 922.13 MHz; Rb^+-FAU at 500 °C under dried N_2 flow (100 mg): Q factor = 1323.23, f_{max} = 922.29 MHz. The calculated real and imaginary parts of the permittivity (ε' and ε'', respectively) and the dielectric loss factor ($\tan\delta$) were summarized in Table S1.

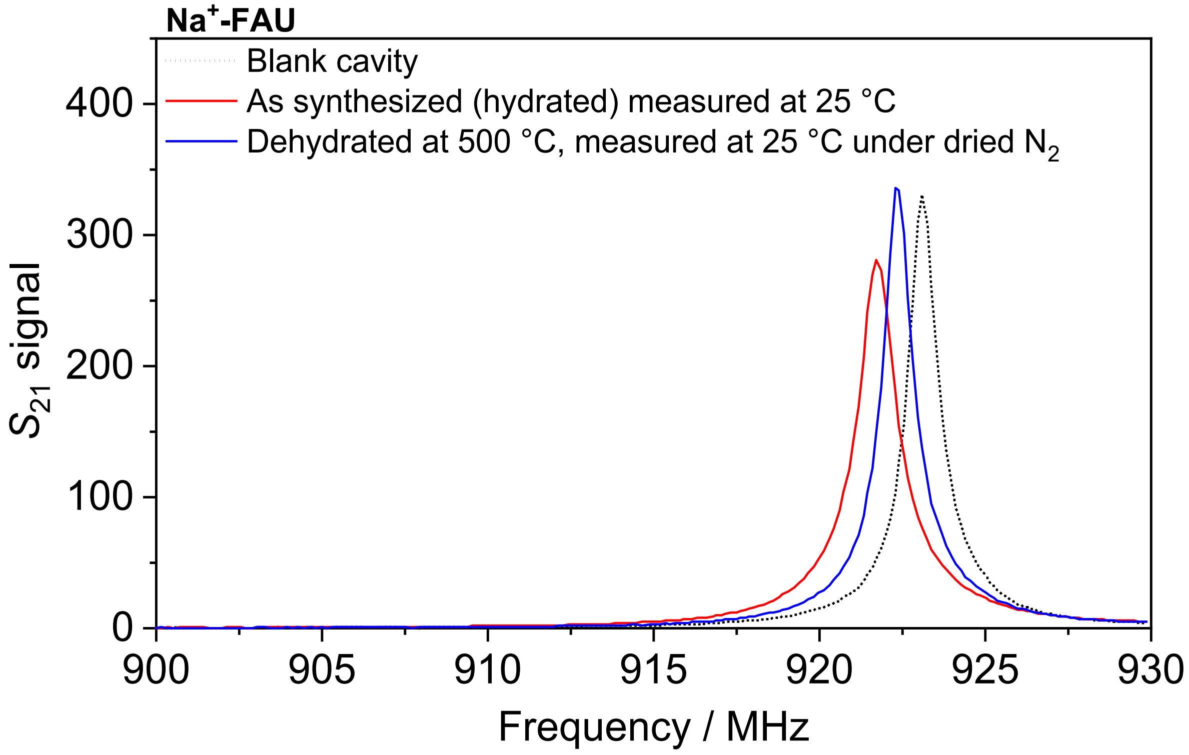

Fig. S8| S_{21} parameter of Na^+-FAU zeolite inserted in TM_{010} cavity. Blank cavity: Q factor = 1567.21, f_{max} = 923.09 MHz; As synthesized Na^+-FAU at 25 °C (100 mg): Q factor = 1160.84, f_{max} = 921.71 MHz; Dehydrated Na^+-FAU at 25 °C under dried N_2 flow (100 mg): Q factor = 1497.22, f_{max} = 922.29 MHz. The calculated real and imaginary parts of the permittivity (ε' and ε'', respectively) and the dielectric loss factor (tanδ) were summarized in Table S1.

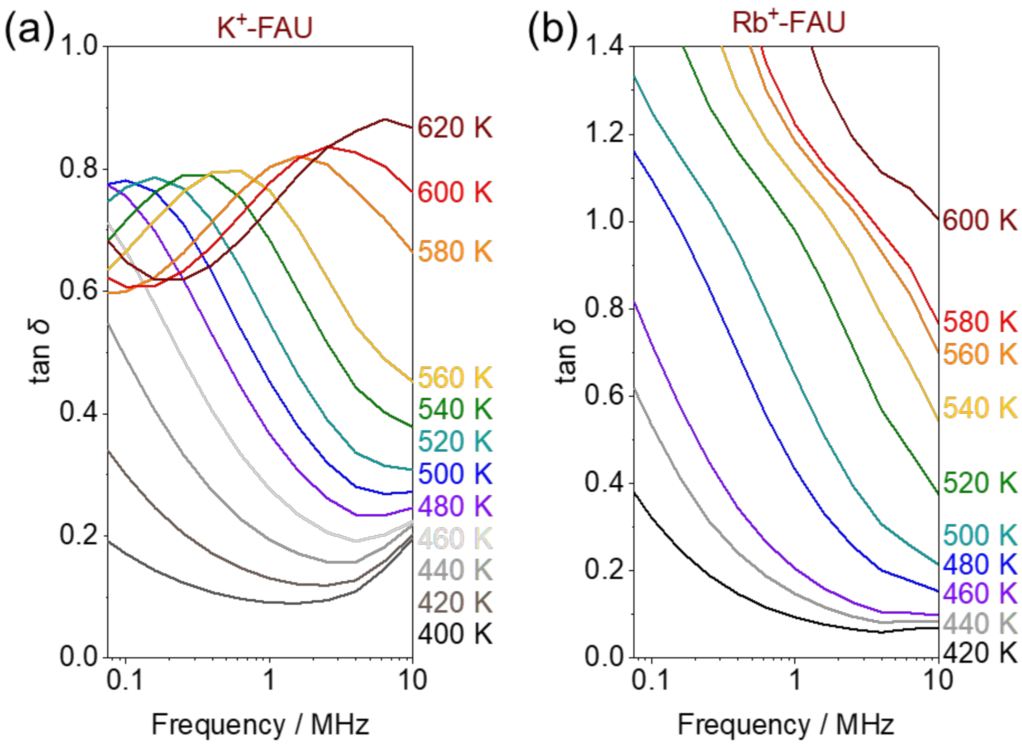

Fig. S9| Dielectric spectra of (a) K^{+}-FAU and (b) Rb^{+}-FAU. The samples were dehydrated at 500 °C before the measurement.

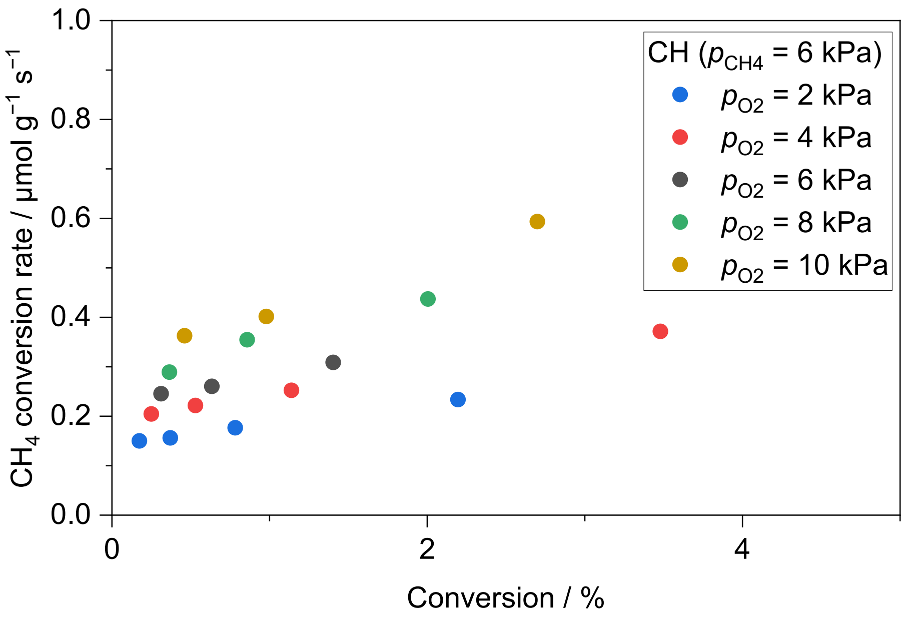

Fig. S10| O_2 partial pressure dependence of Cs^+-FAU zeolite under CH. CH_4 conversion rate over Cs^+-FAU as a function of CH_4 conversion under various O2 partial pressure at 700 °C, CH. Total pressure: 101 kPa. Gas composition: 6 kPa CH_4, 2-10 kPa O_2, N_2 balance. Total flow rate: 20 – 200 mL min^{-1}. Catalyst amount: 100 mg.

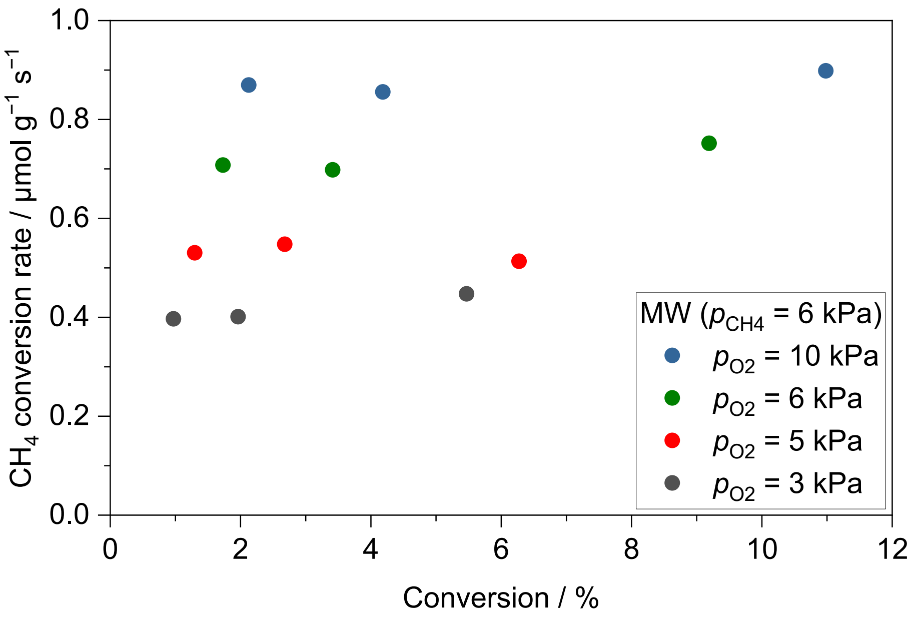

Fig. S11| O_2 partial pressure dependence of Cs^+-FAU zeolite under MW. CH_4 conversion rate over Cs^+-FAU as a function of CH_4 conversion under various O_2 partial pressure at 500 °C, MW. Total pressure: 101 kPa. Gas composition: 6 kPa CH_4, 3-10 kPa O_2, N_2 balance. Total flow rate: 20 – 200 mL min^{-1}. Catalyst amount: 100 mg.

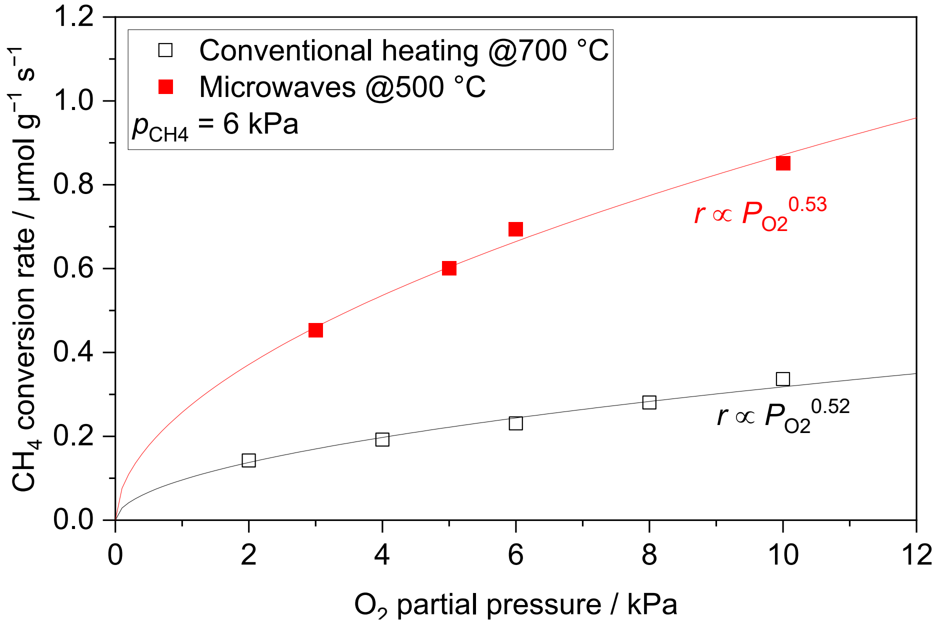

Fig. S12| O_2 partial pressure dependence of CH_4 conversion performance over Cs^+-FAU zeolite. The y-axis represents the zero-conversion extrapolated CH_4 conversion rate. Total pressure: 101 kPa. Gas composition: 6 kPa CH_4 with various partial pressure of O_2, N_2 balance. Catalyst amount: 100 mg. The solid line shows the fitting curve with an O_2 order of 0.53 for MWs and 0.52 for CH.

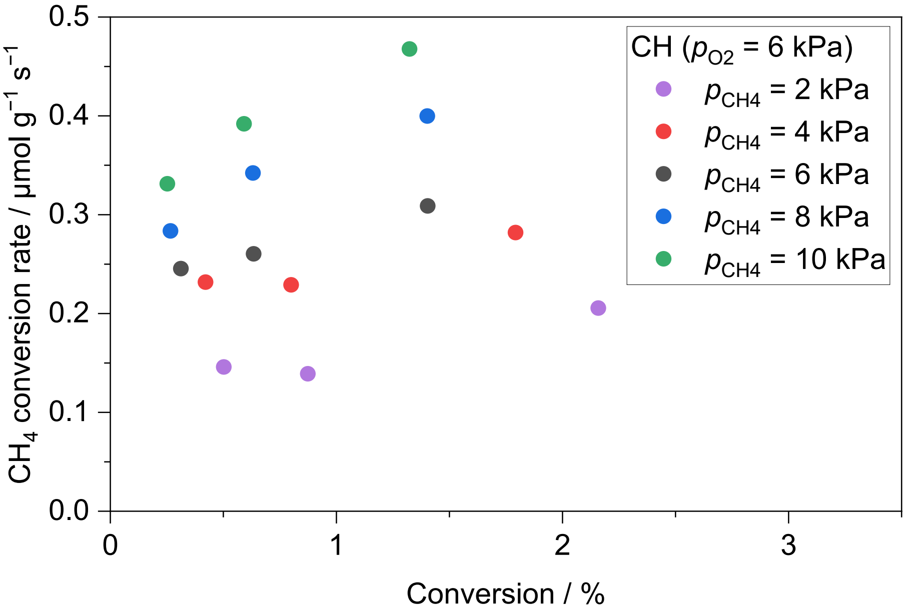

Fig. S13| CH_4 partial pressure dependence of Cs^+-FAU zeolite under CH. CH_4 conversion rate over Cs^+-FAU as a function of CH_4 conversion under various CH_4 partial pressure at 700 °C, CH. Total pressure: 101 kPa. Gas composition: 2-10 kPa CH_4, 6 kPa O_2, N_2 balance. Total flow rate: 20 – 100 mL min^{-1}. Catalyst amount: 100 mg.

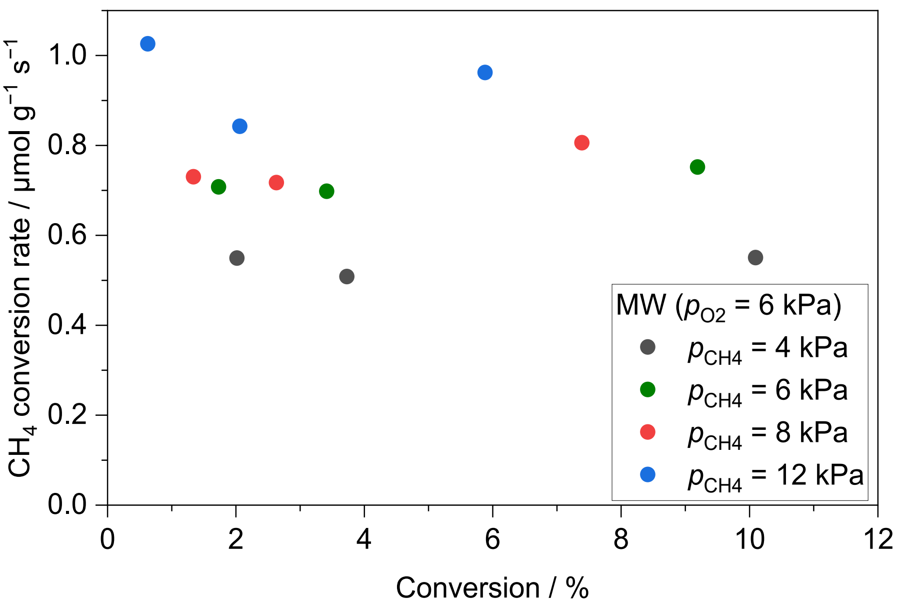

Fig. S14| CH_4 partial pressure dependence of Cs^+-FAU zeolite under MW. CH_4 conversion rate over Cs^+-FAU as a function of CH_4 conversion under various CH_4 partial pressure at 500 °C, MW. Total pressure: 101 kPa. Gas composition: 4-12 kPa CH_4, 6 kPa O_2, N_2 balance. Total flow rate: 20 – 100 mL min^{-1}. Catalyst amount: 100 mg.

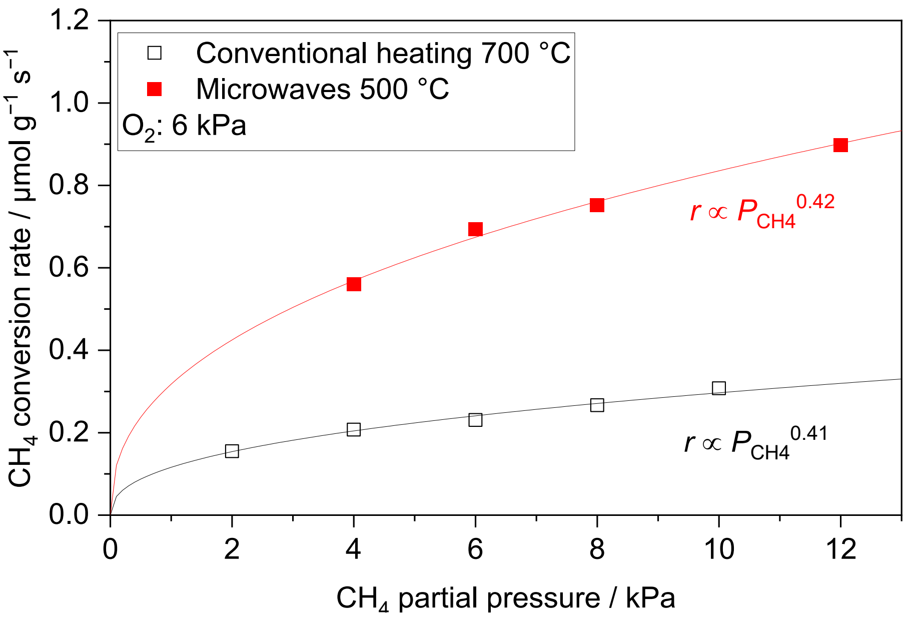

Fig. S15| CH_4 partial pressure dependence of CH_4 conversion performance over Cs^+-FAU zeolite. The y-axis represents the zero-conversion extrapolated CH_4 conversion rate. Total pressure: 101 kPa. Gas composition: 6 kPa O_2 with various partial pressure of CH_4, N_2 balance. Catalyst amount: 100 mg. The solid line shows the fitting curve with a CH_4 order of 0.42 for MWs and 0.41 for CH.

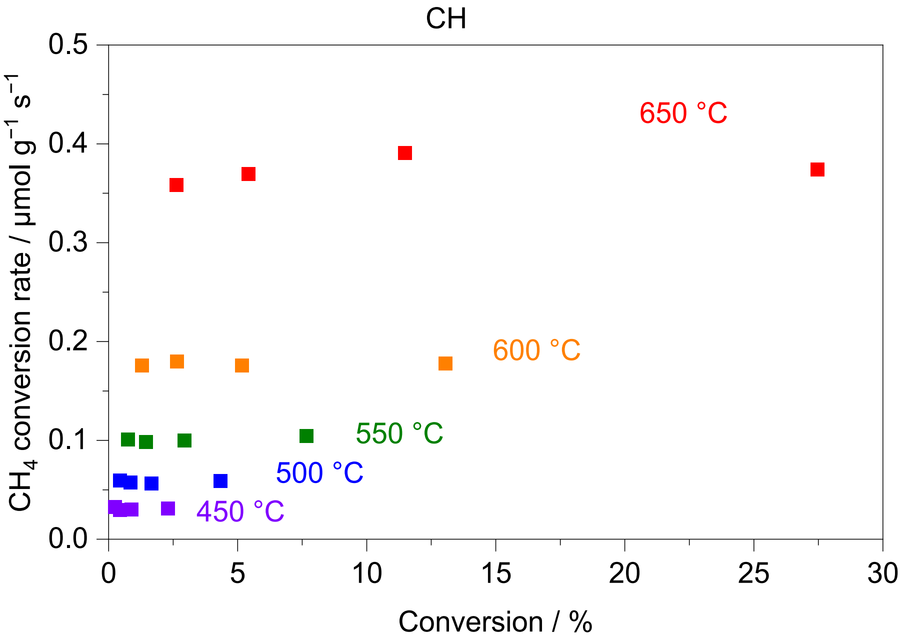

Fig. S16| CO oxidation performance of Cs^+-FAU zeolite under CH. CO conversion rate over Cs^+-FAU as a function of CO conversion under CH at various temperatures. Total pressure: 101 kPa. Gas composition: 1 kPa CO and 6 kPa O_2, N_2 balance. Total flow rate: 20 – 200 mL min^{-1}. Catalyst amount: 100 mg.

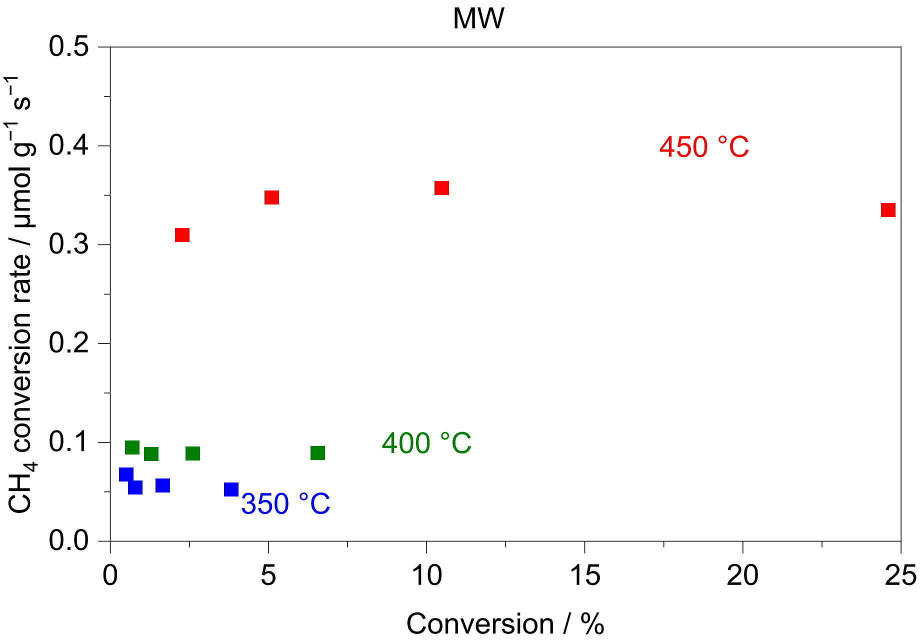

Fig. S17| CO oxidation performance of Cs^{+}-FAU zeolite under MW. CO conversion rate over Cs^{+}-FAU as a function of CO conversion under MW at various temperatures. Total pressure: 101 kPa. Gas composition: 1 kPa CO and 6 kPa O_2, N_2 balance. Total flow rate: 20 – 200 mL min^{-1}. Catalyst amount: 100 mg.

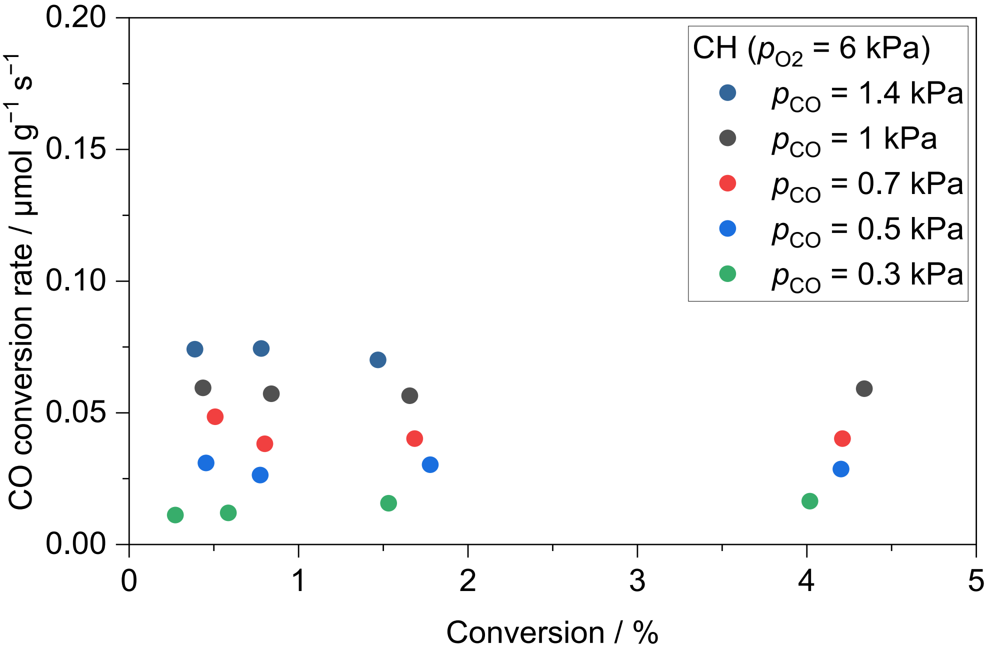

Fig. S18| CO partial pressure dependence of Cs^+-FAU zeolite under CH. CO conversion rate over Cs^+-FAU as a function of CO conversion under various CO partial pressure at 500 °C, CH. Total pressure: 101 kPa. Gas composition: 0.3-1.4 kPa CO, 6 kPa O_2, N_2 balance. Total flow rate: 20 – 200 mL min^{-1}. Catalyst amount: 100 mg.

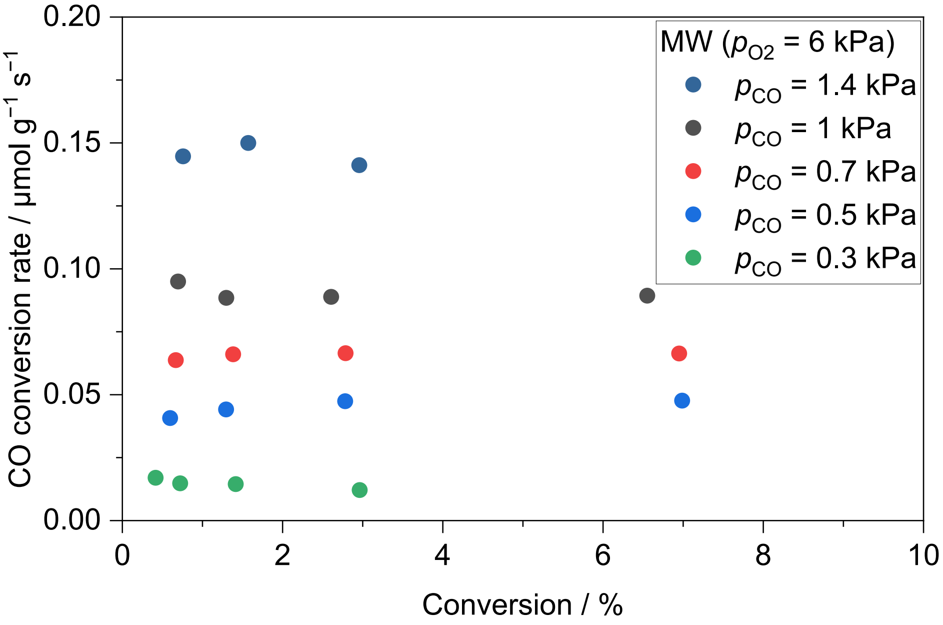

Fig. S19| CO partial pressure dependence of Cs^+-FAU zeolite under MW. CO conversion rate over Cs^+-FAU as a function of CO conversion under various CO partial pressure at 400 °C, MW. Total pressure: 101 kPa. Gas composition: 0.3-1.4 kPa CO, 6 kPa O_2, N_2 balance. Total flow rate: 20 – 200 mL min^{-1}. Catalyst amount: 100 mg.

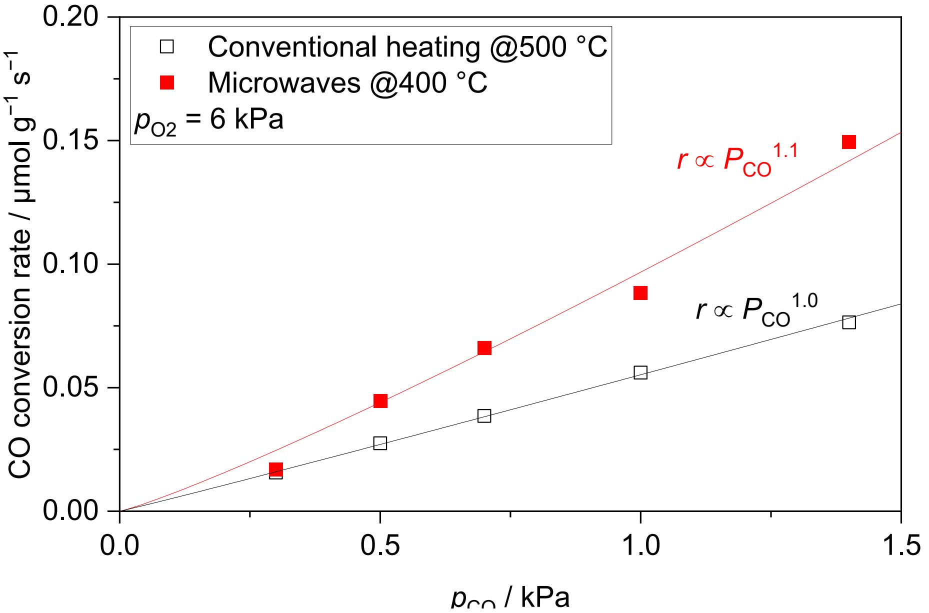

Fig. S20| CO partial pressure dependence of CO conversion performance over Cs^{+}-FAU zeolite. y-axis represents the zero-conversion extrapolated CO conversion rate. Total pressure: 101 kPa. Gas composition: 6 kPa O_2 with various partial pressure of CO, N_2 balance. Catalyst amount: 100 mg. The solid line shows the fitting curve with a CO order of 1.1 for MWs and 1.0 for CH.

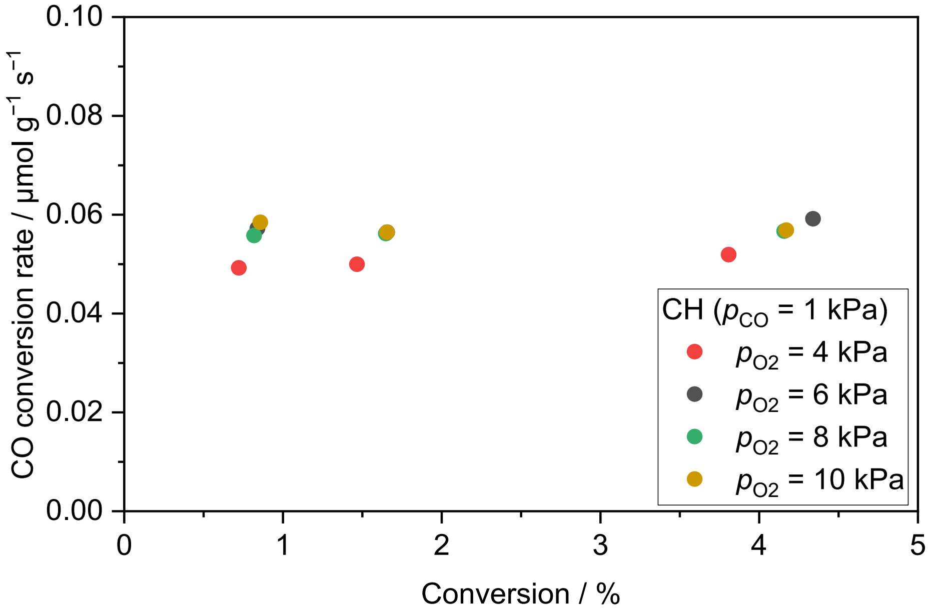

Fig. S21| O_2 partial pressure dependence of Cs^+-FAU zeolite under CH. CO conversion rate over Cs^+-FAU as a function of CO conversion under various O_2 partial pressure at 500 °C, CH. Total pressure: 101 kPa. Gas composition: 1 kPa CO, 4-10 kPa O_2, N_2 balance. Total flow rate: 20 – 200 mL min^{-1}. Catalyst amount: 100 mg.

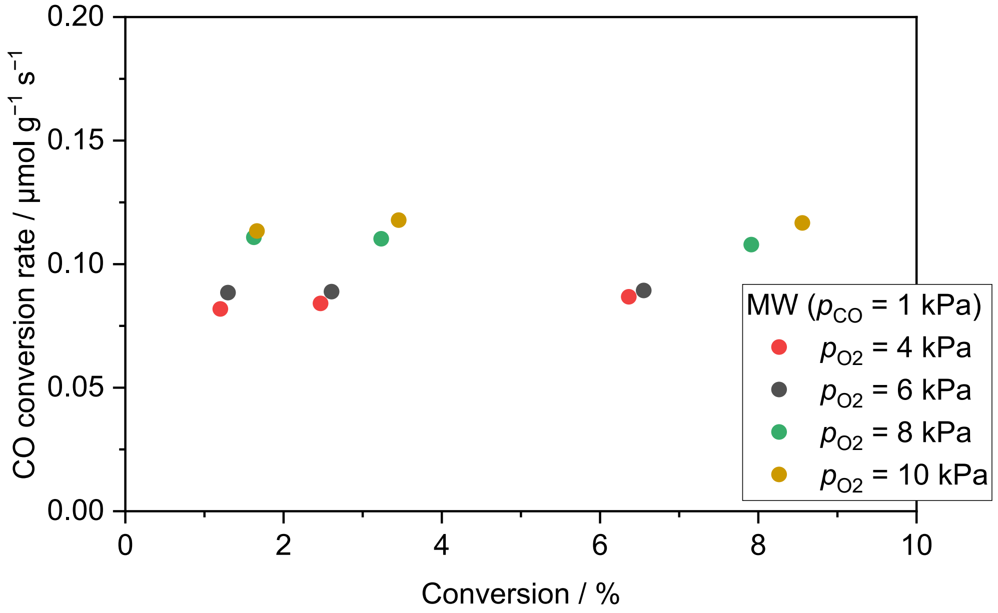

Fig. S22| O_2 partial pressure dependence of Cs^+-FAU zeolite under MW. CO conversion rate over Cs^+-FAU as a function of CO conversion under various O_2 partial pressure at 500 °C, CH. Total pressure: 101 kPa. Gas composition: 1 kPa CO, 4-10 kPa O_2, N_2 balance. Total flow rate: 20 – 200 mL min^{-1}. Catalyst amount: 100 mg.

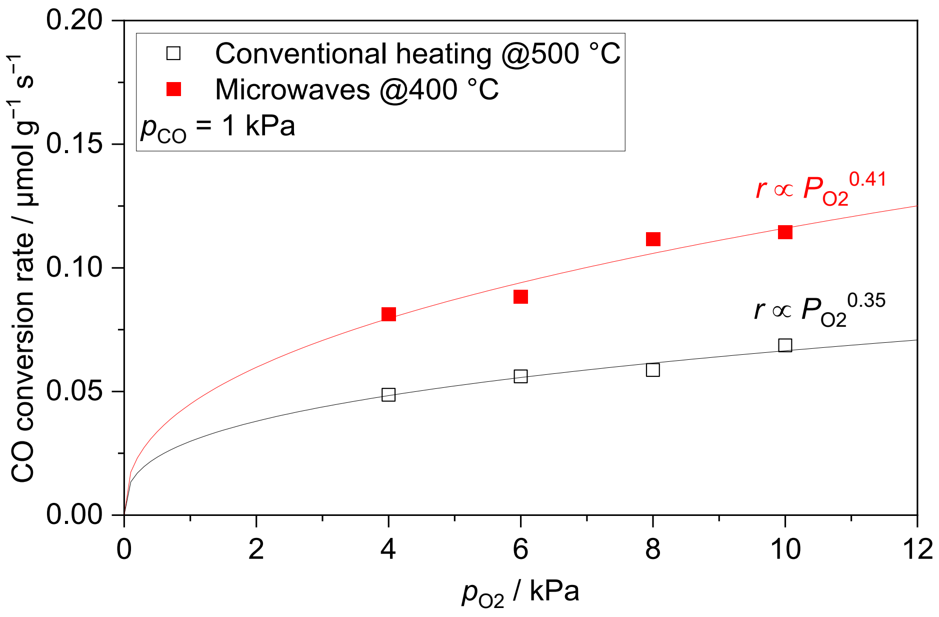

Fig. S23| O_2 partial pressure dependence of CO conversion performance over Cs^+-FAU zeolite. The y-axis represents the zero-conversion extrapolated CO conversion rate. Total pressure: 101 kPa. Gas composition: 1 kPa CO with various partial pressure of O_2, N_2 balance. Catalyst amount: 100 mg. The solid line shows the fitting curve with an O_2 order of 0.41 for MWs and 0.35 for CH.

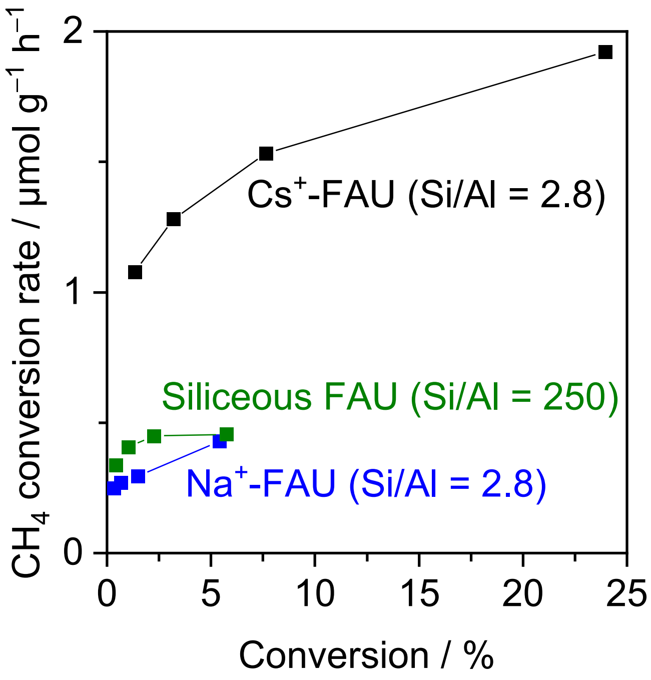

Fig. S24| CH_4 conversion rate of other zeolites under CH. 750 °C. Total pressure: 101 kPa. Gas composition: 6 kPa CH_4 and 6 kPa O_2, N_2 balance. Total flow rate: 20 – 200 mL min^{-1}. Catalyst amount: 100 mg.

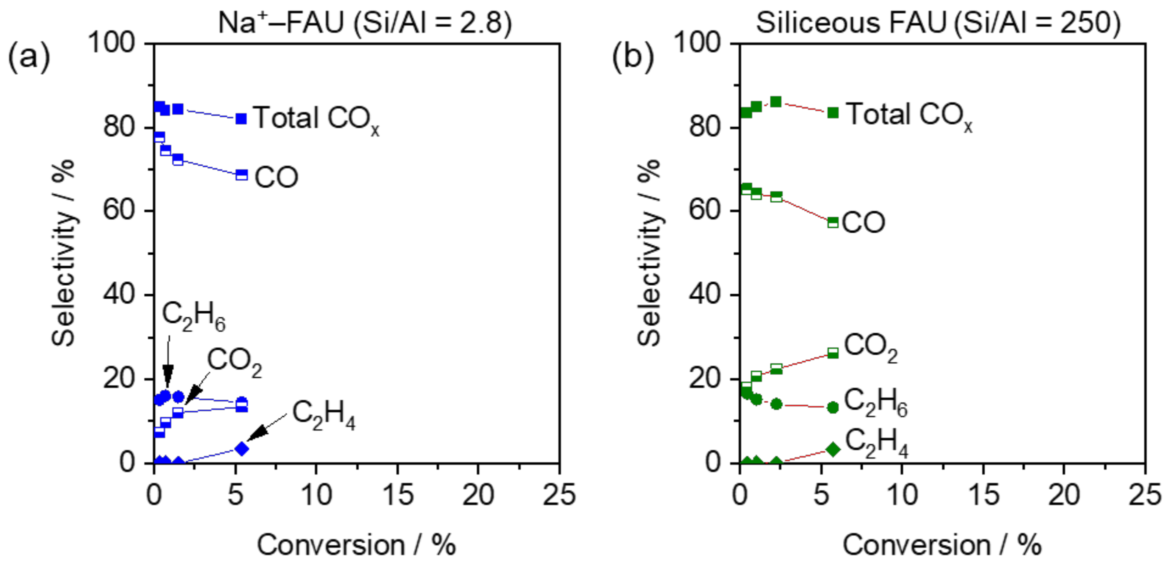

Fig. S25| Carbon selectivity of other zeolites under CH. 750 °C. Total pressure: 101 kPa. Gas composition: 6 kPa CH_4 and 6 kPa O_2, N_2 balance. Total flow rate: 20 – 200 mL min^{-1}. Catalyst amount: 100 mg.

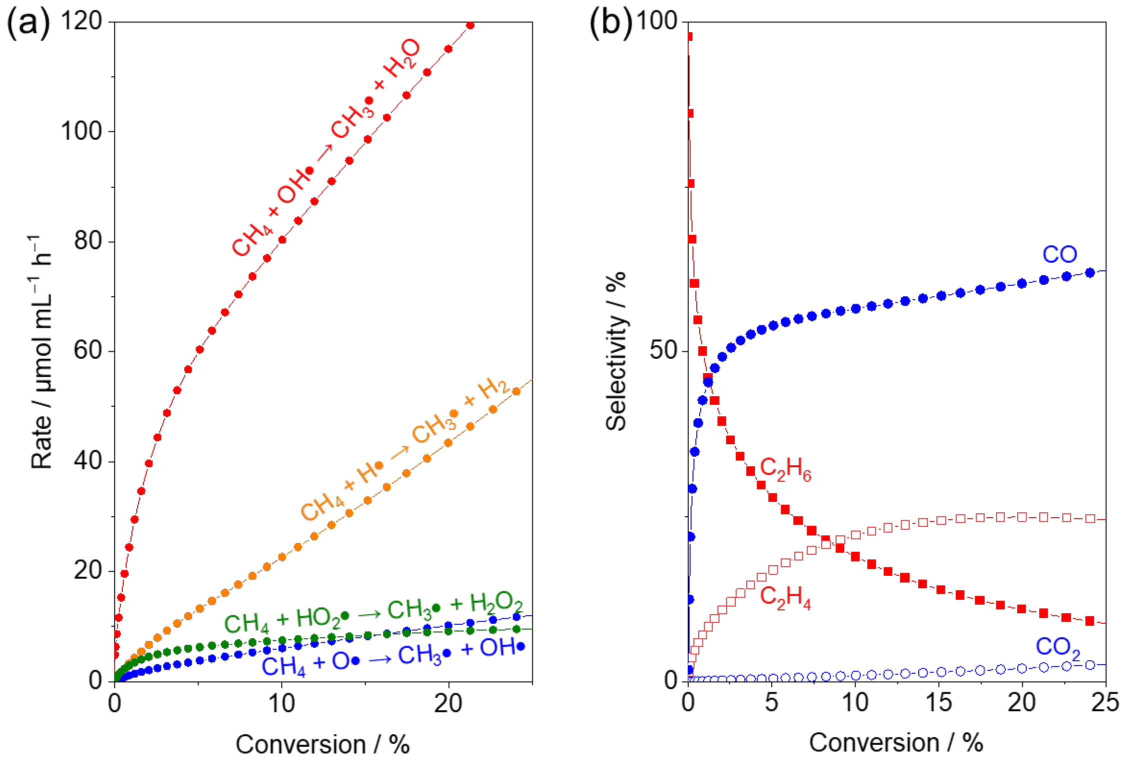

Fig. S26 Simulated gas phase CH_4 oxidation kinetics. (a) CH_4 oxidation rate through four major pathways and (b) product selectivity as a function of CH_4 conversion. 750 °C. Gas composition: 6 kPa CO, 6 kPa O_2, N_2 balance.

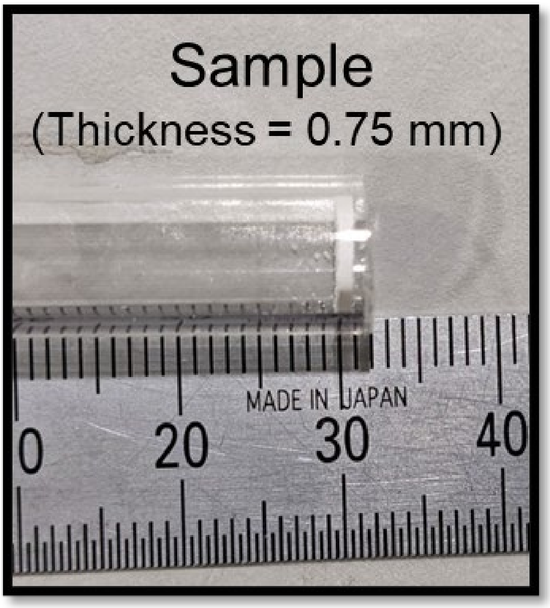

Fig. S27| Photograph of specimen for MW in situ synchrotron spectroscopy. Pelletized Cs^+-FAU disk with a thickness of 0.75 mm was fixed in the quartz tube (internal diameter = 7 mm).

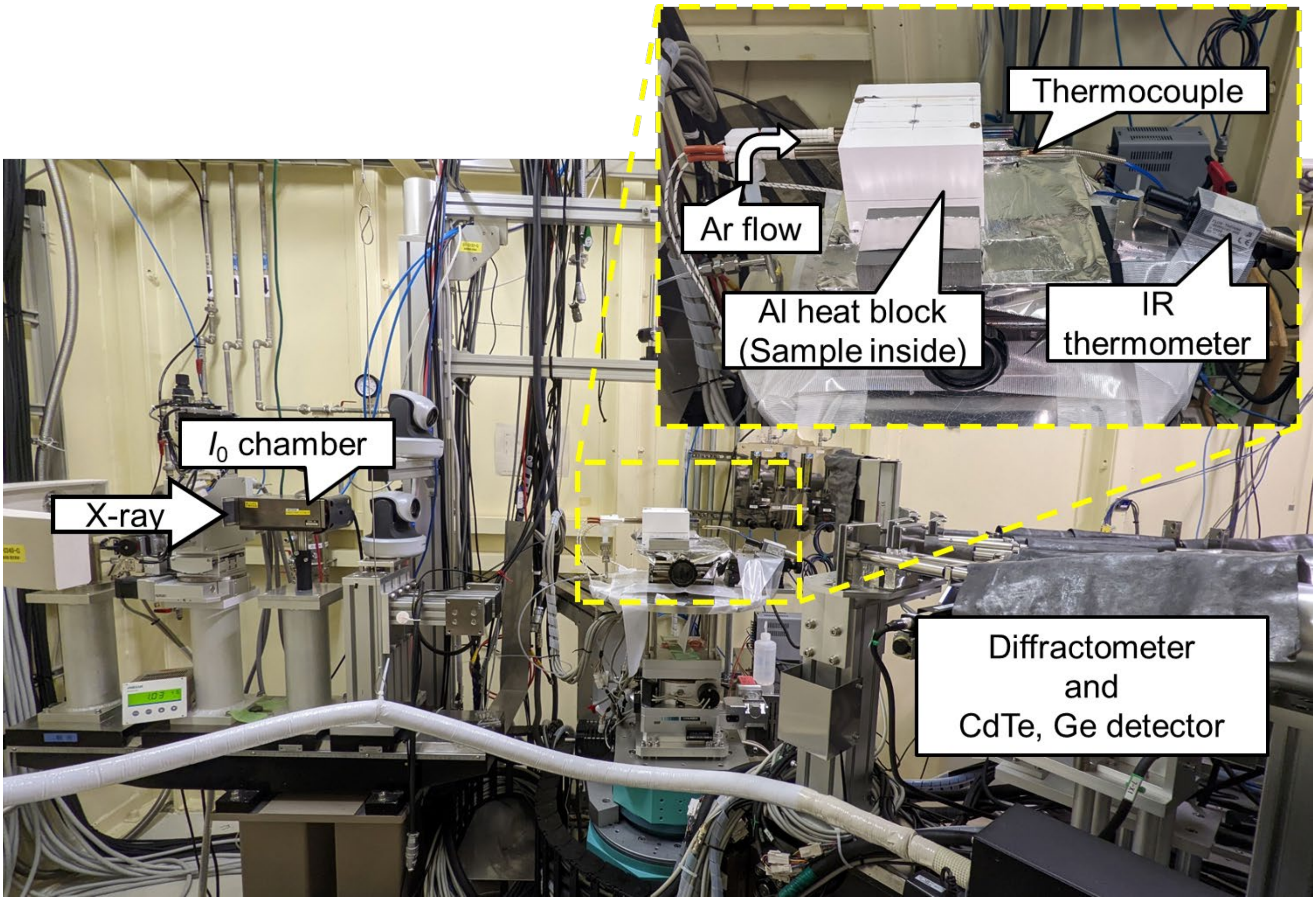

Fig. S28| Photograph of setup for in situ high energy X-ray total scattering measurement under CH.

The measurement was conducted at beamline BL04B2 at SPring-8, Hyogo, Japan. The sample specimen shown in Figure S27 was placed in the aluminum-made heating block. The sample temperature was controlled by a K-type thermocouple inserted into the heating block. The IR thermometer monitored the temperature at the center of the circular surface of the disk-shaped sample for consistency with experiments under MW irradiation.

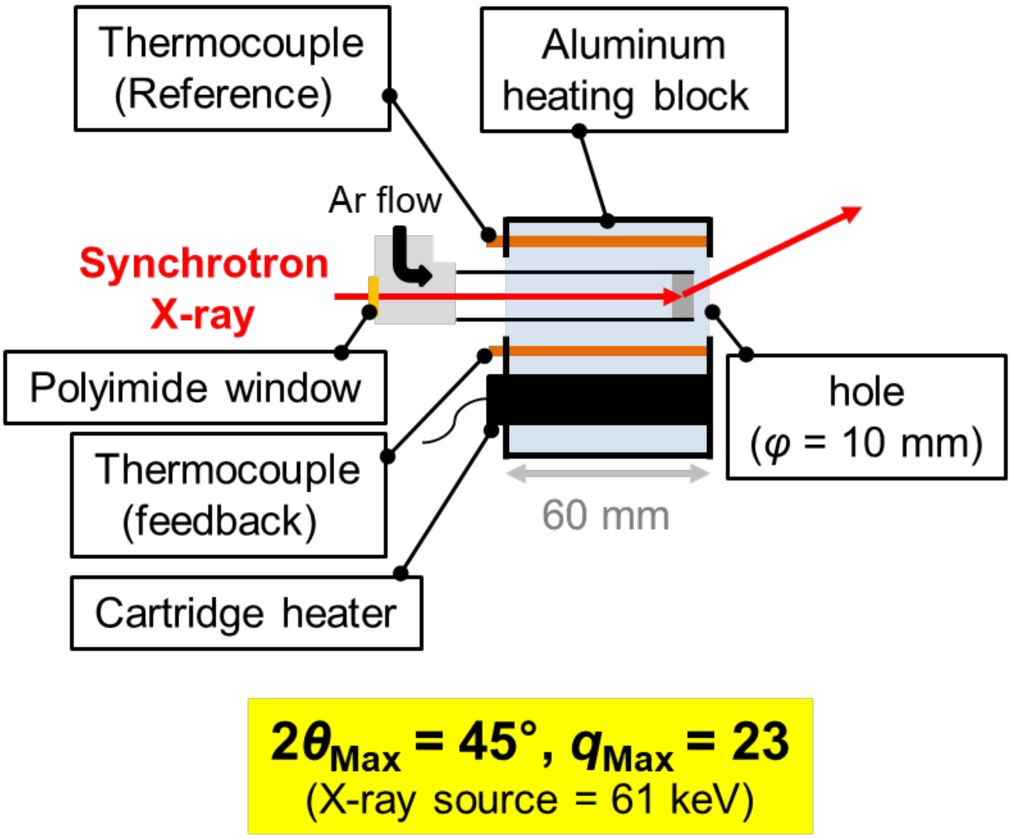

Fig. S29| Schematic illustration of in situ high energy X-ray total scattering measurement under CH.

Maximum 2θ ($2\theta_{Max}$) was achieved as 45°, which corresponds to a maximum q (q_{Max}) of 23 Å^{-1} with X-ray energy of 61 keV ($q = 4\pi\sin\theta/\lambda$). Dry Ar gas (50 mL min^{-1}) was fed to the specimen to keep the dehydrated condition.

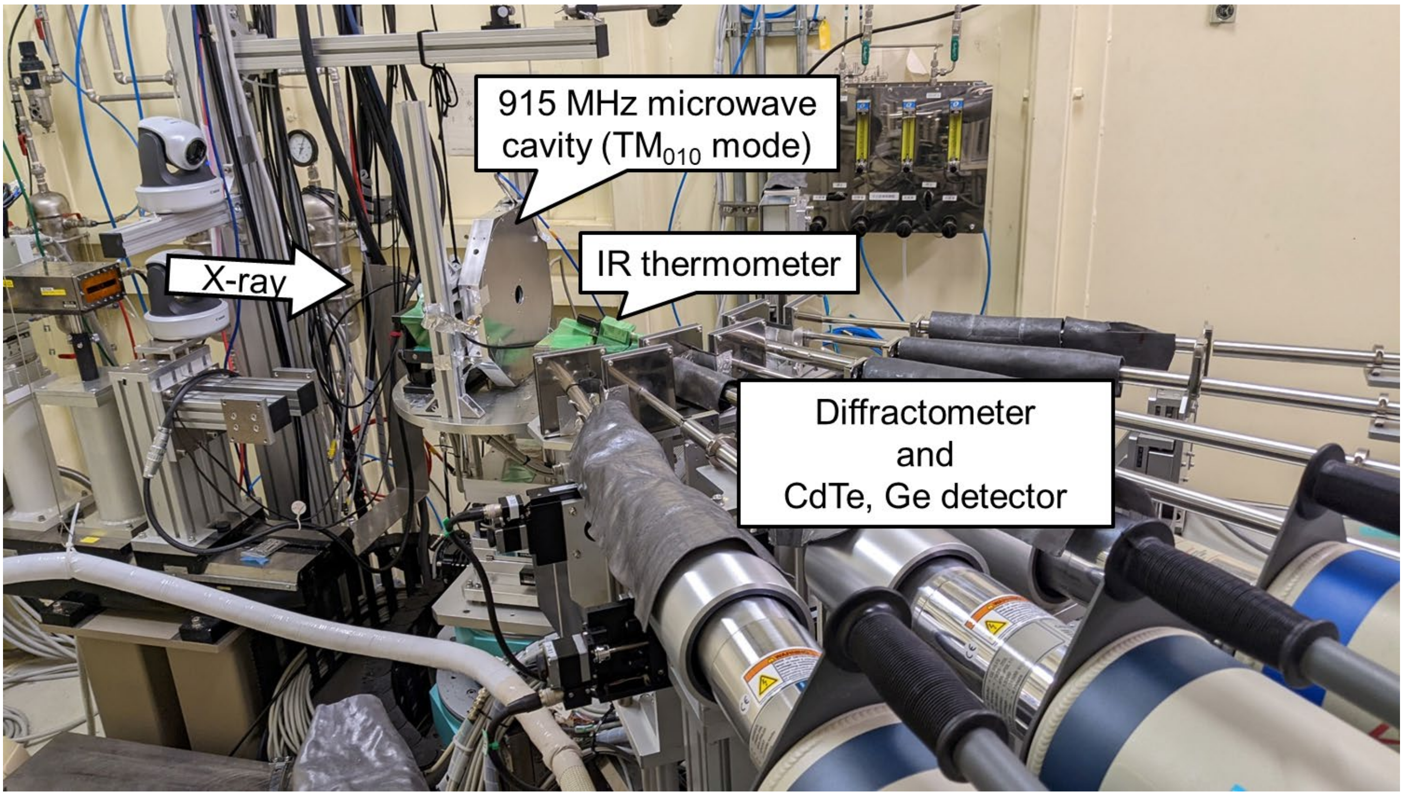

Fig. S30| Photograph of setup for in situ high energy X-ray total scattering measurement under MWs.

The sample specimen shown in Figure S27 was placed at the center of the 915 MHz MW cavity. The temperature at the center of the circular surface of the disk-shaped sample was measured by an infrared thermometer. The spot for temperature measurement was a φ = 4 mm diameter circle, and the X-ray beam spot was 2 × 2 mm square.

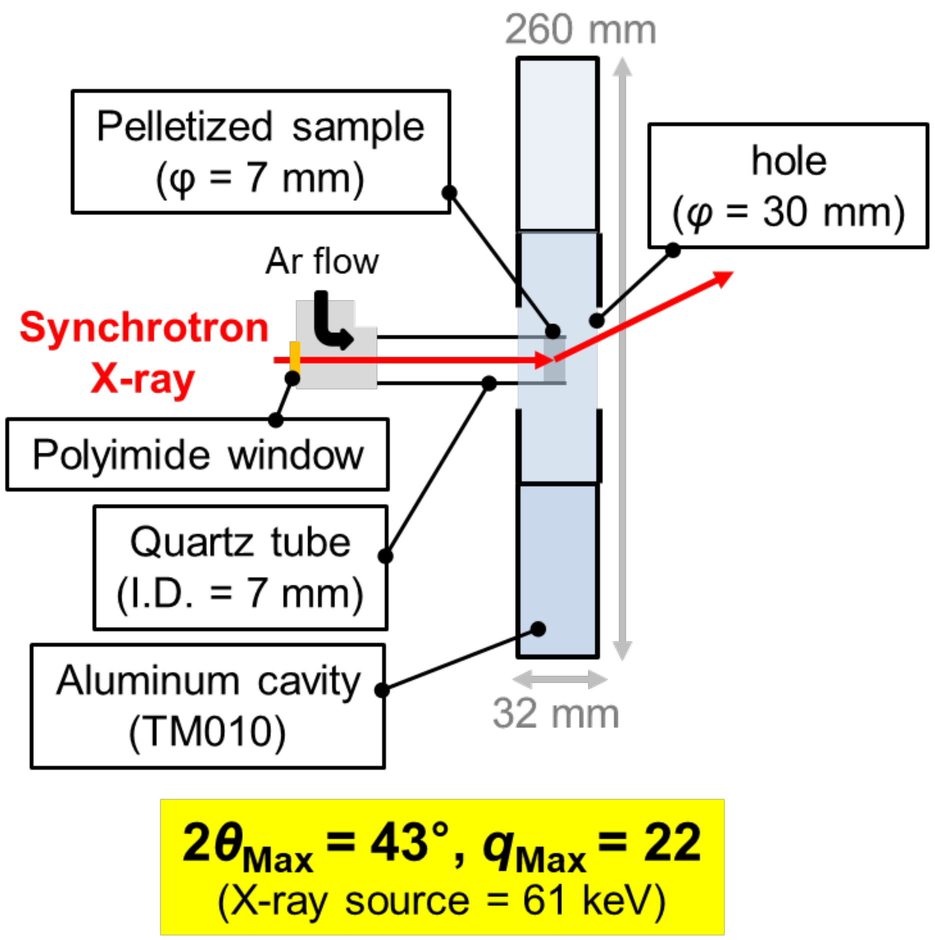

Fig. S31| Schematic illustration of in situ high energy X-ray total scattering measurement under MWs.

Maximum 2θ ($2\theta_{Max}$) was achieved as 43°, which corresponds to a maximum q (q_{Max}) of 22 Å^{-1} with X-ray energy of 61 keV ($q = 4\pi\sin\theta/\lambda$). Dry Ar gas (50 mL min^{-1}) was fed to the specimen to keep the dehydrated condition.

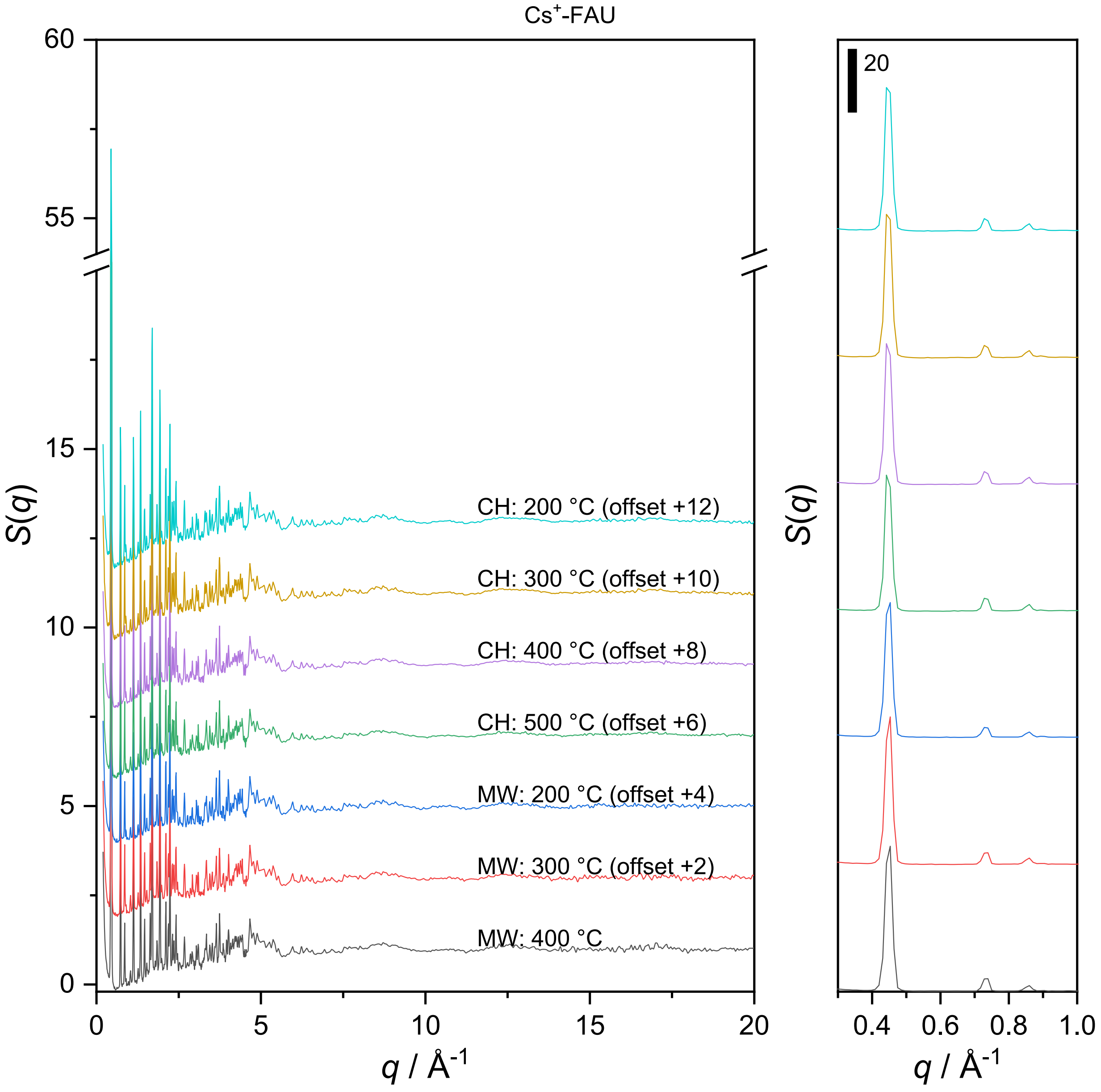

Fig. S32| Total scattering factor, $S(q)$, of Cs^+-FAU under MWs or CH. The q_{max} collected was 20 $Å^{-1}$.

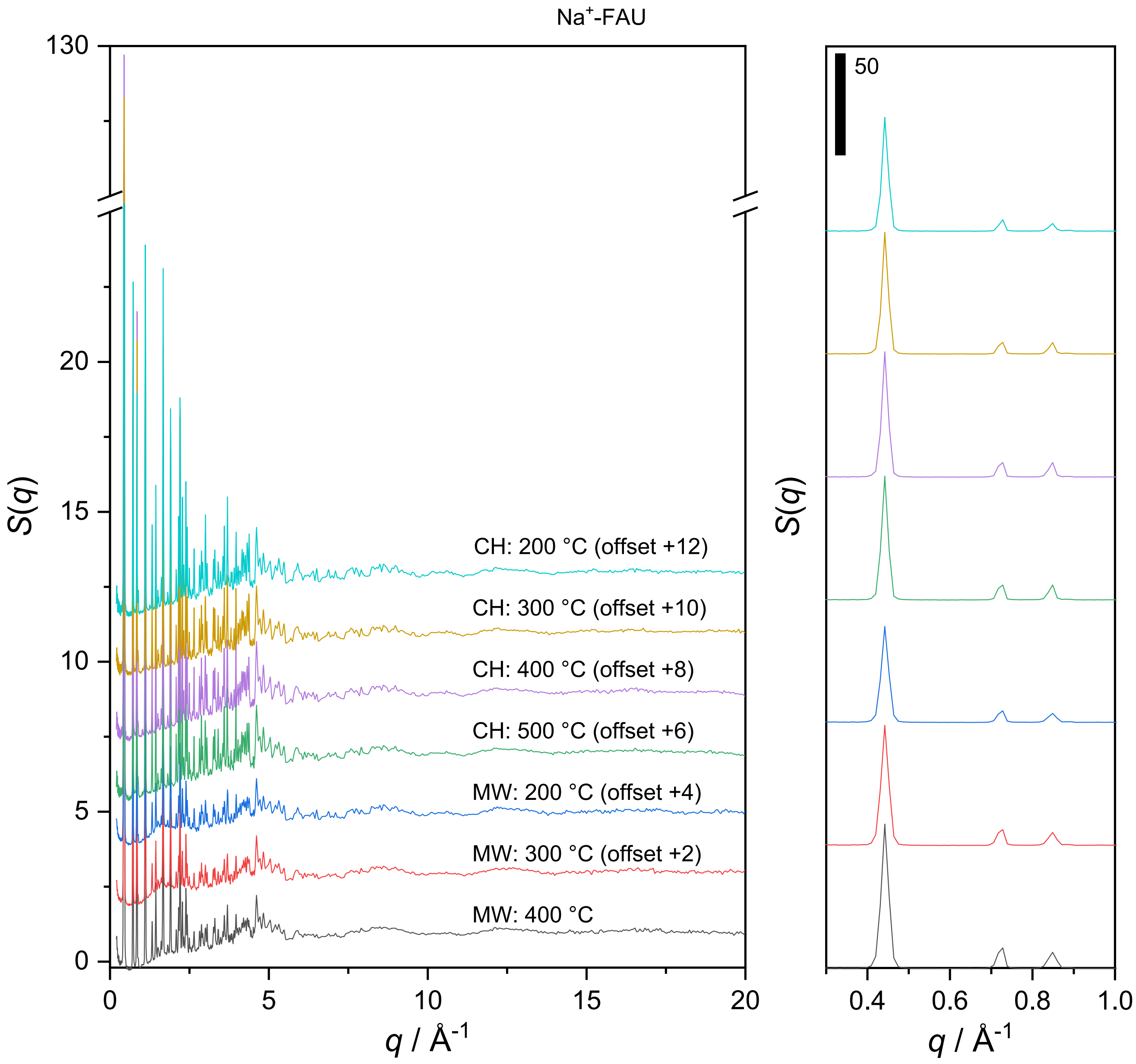

Fig. S33| Total scattering factor, $S(q)$, of Na^+-FAU under MWs or CH. The q_{max} collected was 20 $Å^{-1}$.

Simulated PDF from MD calculation.

The shifts in pairs other than Cs–O and Na–O also showed good agreement with the experimental results (Figure S34 and S35). Both a long–distance shift in the Cs–Al pair at 3.8 Å under CH (Figure S34(a)) and a shorter–distance shift in the Cs–Si pair at 3.8 Å under MWs (Figure S34(c)) were consistent with the experimental results in Figure 3(a). Slight shifts in the Si–O and Al–O pair at 3.5 ~ 4.5 Å (Figure S34(b)(d) and S36(b)(d)) under both CH and MWs explained the experimental results of both Cs^+– and Na^+–FAU. However, the peak shift in the Na–Al and Na–Si pair (Figure S35(a) and (b)) expected by MD calculation was not shown in the experimental results, probably due to the low atomic scattering factor on Na^+.

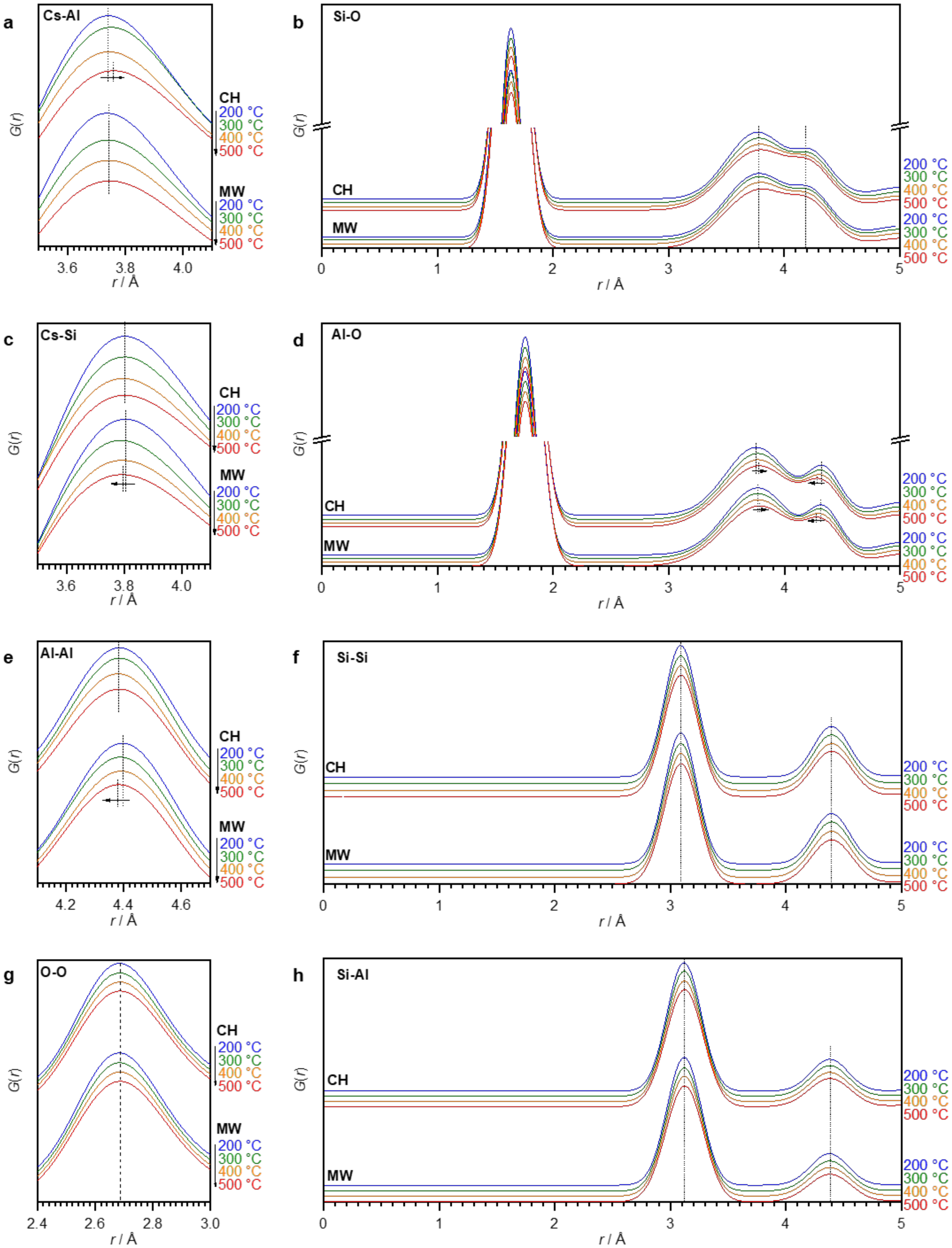

a
Cs-Al
G(r)
CH
200 °C
300 °C
400 °C
500 °C
MW
200 °C
300 °C
400 °C
500 °C
3.6
3.8
4.0
r / Å
b
Si-O
G(r)
CH
MW
200 °C
300 °C
400 °C
500 °C
200 °C
300 °C
400 °C
500 °C
0
1
2
3
4
5
r / Å
c
Cs-Si
G(r)
CH
200 °C
300 °C
400 °C
500 °C
MW
200 °C
300 °C
400 °C
500 °C
3.6
3.8
4.0
r / Å
d
Al-O
G(r)
CH
MW
200 °C
300 °C
400 °C
500 °C
200 °C
300 °C
400 °C
500 °C
0
1
2
3
4
5
r / Å
e
Al-Al
G(r)
CH
200 °C
300 °C
400 °C
500 °C
MW
200 °C
300 °C
400 °C
500 °C
4.2
4.4
4.6
r / Å
f
Si-Si
G(r)
CH
MW
200 °C
300 °C
400 °C
500 °C
200 °C
300 °C
400 °C
500 °C
0
1
2
3
4
5
r / Å
g
O-O
G(r)
CH
200 °C
300 °C
400 °C
500 °C
MW
200 °C
300 °C
400 °C
500 °C
2.4
2.6
2.8
3.0
r / Å
h
Si-Al
G(r)
CH
MW
200 °C
300 °C
400 °C
500 °C
200 °C
300 °C
400 °C
500 °C
0
1
2
3
4
5
r / Å

Fig. S34| Simulated PDF results of Cs^+-FAU from MD calculation. Na atoms of the crystal structure of the currently utilized HSZ-320NAA zeolite were replaced with Cs atoms to create the initial structure of the molecular dynamics simulations. To mimic the local heating of Cs^+ cations in the FAU zeolite cavity under MW irradiation, Cs atoms were heated to 350, 475, 630, and 750 °C while the temperature of the aluminosilicate structure was kept under 200, 300, 400, and 500 °C, respectively. For the CH condition, equilibrium molecular dynamics runs with the NVT ensemble under 200, 300, 400, and 500 °C were performed.

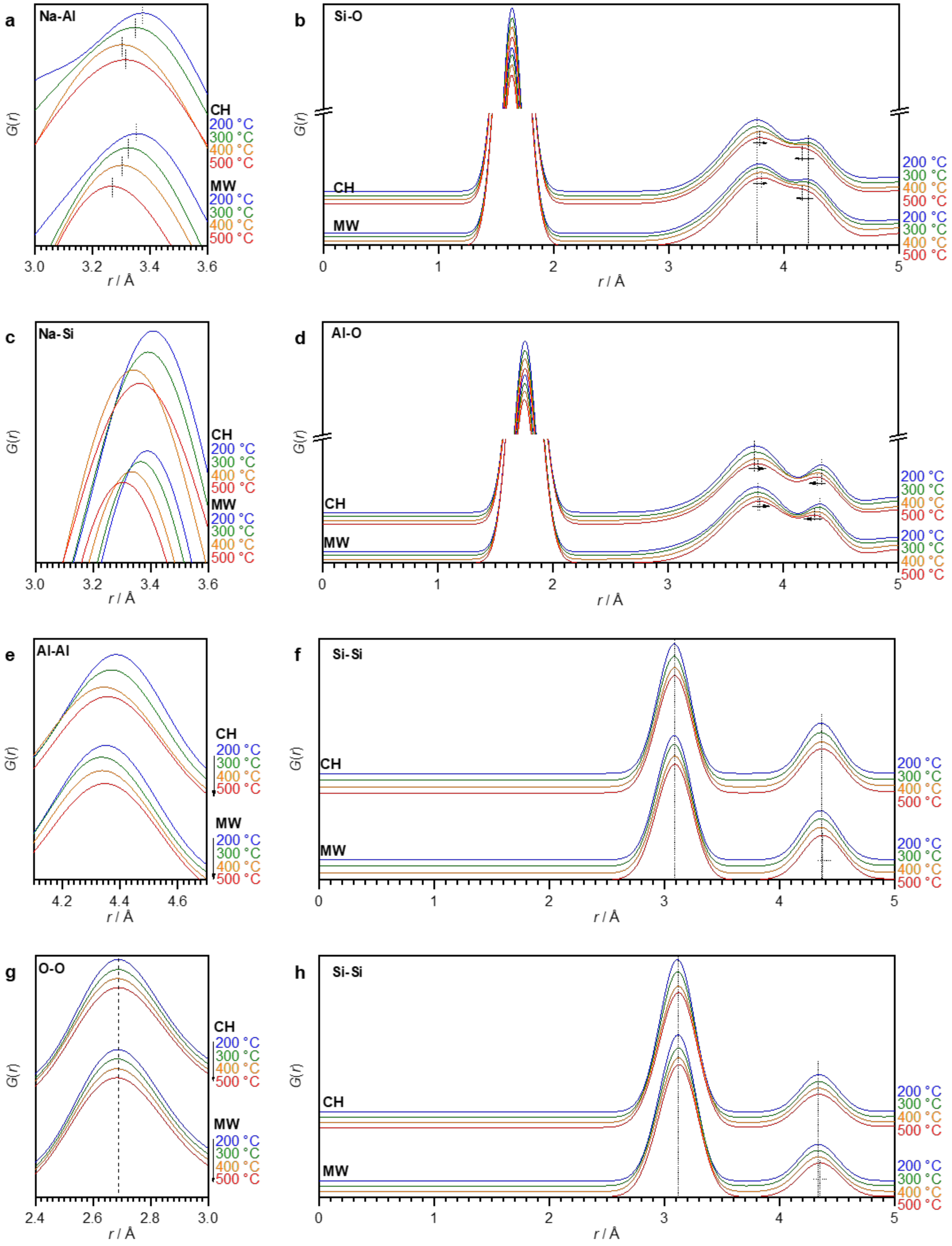

a
Na-Al
G(r)
CH
200 °C
300 °C
400 °C
500 °C
MW
200 °C
300 °C
400 °C
500 °C
3.0
3.2
3.4
3.6
r / Å
b
Si-O
CH
MW
0
1
2
3
4
5
c
Na-Si
d
Al-O
e
Al-Al
4.2
4.4
4.6
f
Si-Si
g
O-O
2.4
2.6
2.8
3.0
h
Si-Si

Fig. S35| Simulated PDF results of Na^+-FAU from MD calculation. The crystal structure of the currently utilized HSZ-320NAA zeolite was employed as the initial structure of the molecular dynamics simulations. To mimic the local heating of Na^+ cations in the FAU zeolite cavity under MW irradiation, Na atoms were heated to 350, 475, 630, and 750 °C while the temperature of the aluminosilicate structure was kept under 200, 300, 400, and 500 °C, respectively. For the CH condition, the equilibrium molecular dynamics runs with the NVT ensemble under 200, 300, 400, and 500 °C were performed.

in situ Cs K–edge XAS measurements

Cs K–edge XAS measurements were also performed to support the local heating of Cs^+ in the zeolite. There is no characteristic change in X–ray absorption near–edge spectra (XANES) by MW irradiation (Figure S37). The extended X–ray absorption fine structure (EXAFS) and Fourier transformed EXAFS (FT–EXAFS) were shown in Figure S38 and 39, respectively. FT–EXAFS of Cs^+–FAU at an almost similar temperature under CH and MWs were compared in Figure S40. Compared to CH, the peak intensity was drastically attenuated by MW heating at ~500 °C, probably due to the Debye–Waller factor induced by the thermal vibration of selectively heated Cs atoms. Thus, the Cs K–edge XAS results support the MW selective heating of the single Cs cation in the zeolite cavity. The relatively lower attenuation of the peak at a longer distance attributed to the Cs–T pair was due to weak EXAFS oscillation of the Cs K–edge, making it difficult to detect long–range peaks.

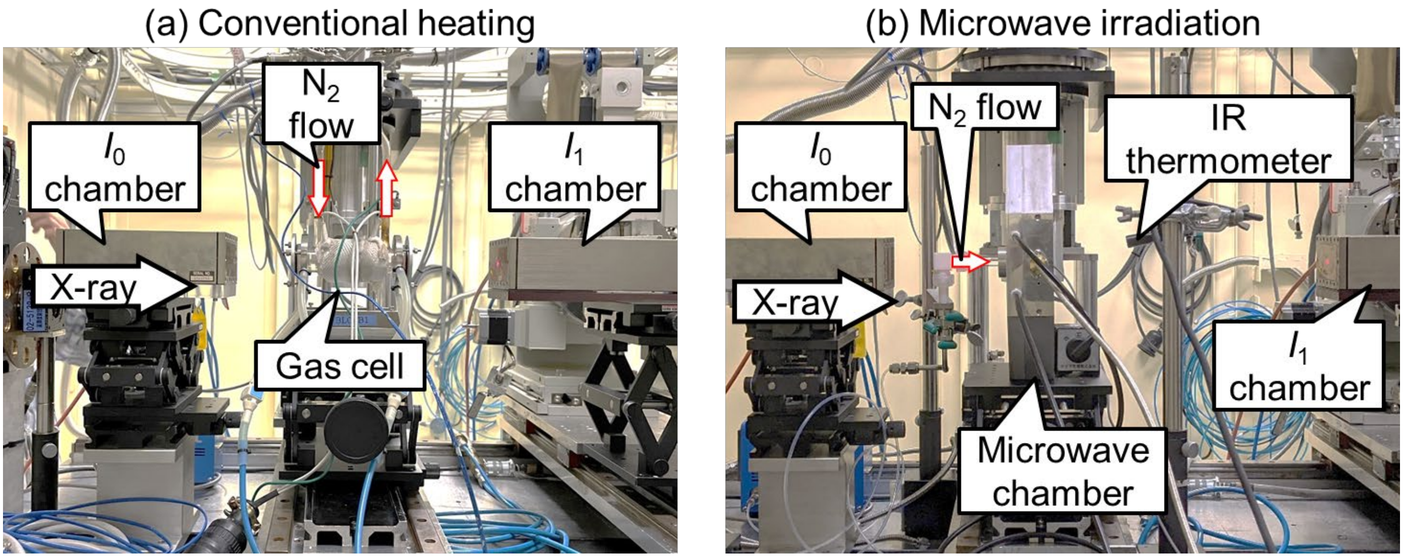

Fig. S36| Photograph of setup for in situ X-ray absorption measurement under (a) CH and (b) MWs.

The measurement was conducted at beamline BL01B1 at SPring-8, Hyogo, Japan. For CH, the sample specimen shown in Figure S31 was placed in a quartz-made gas cell filled with dry N_2 (200 mL min^{-1}). The sample temperature was controlled by a K-type thermocouple inserted into a quartz-made gas cell. For MW irradiation, the sample specimen was placed at the center of the MW chamber. The temperature at the center of the circular surface of the disk-shaped sample was measured by an infrared thermometer. The spot for temperature measurement was a φ = 4 mm diameter circle, and the X-ray beam spot was approximately 0.5 × 3 mm rectangular.

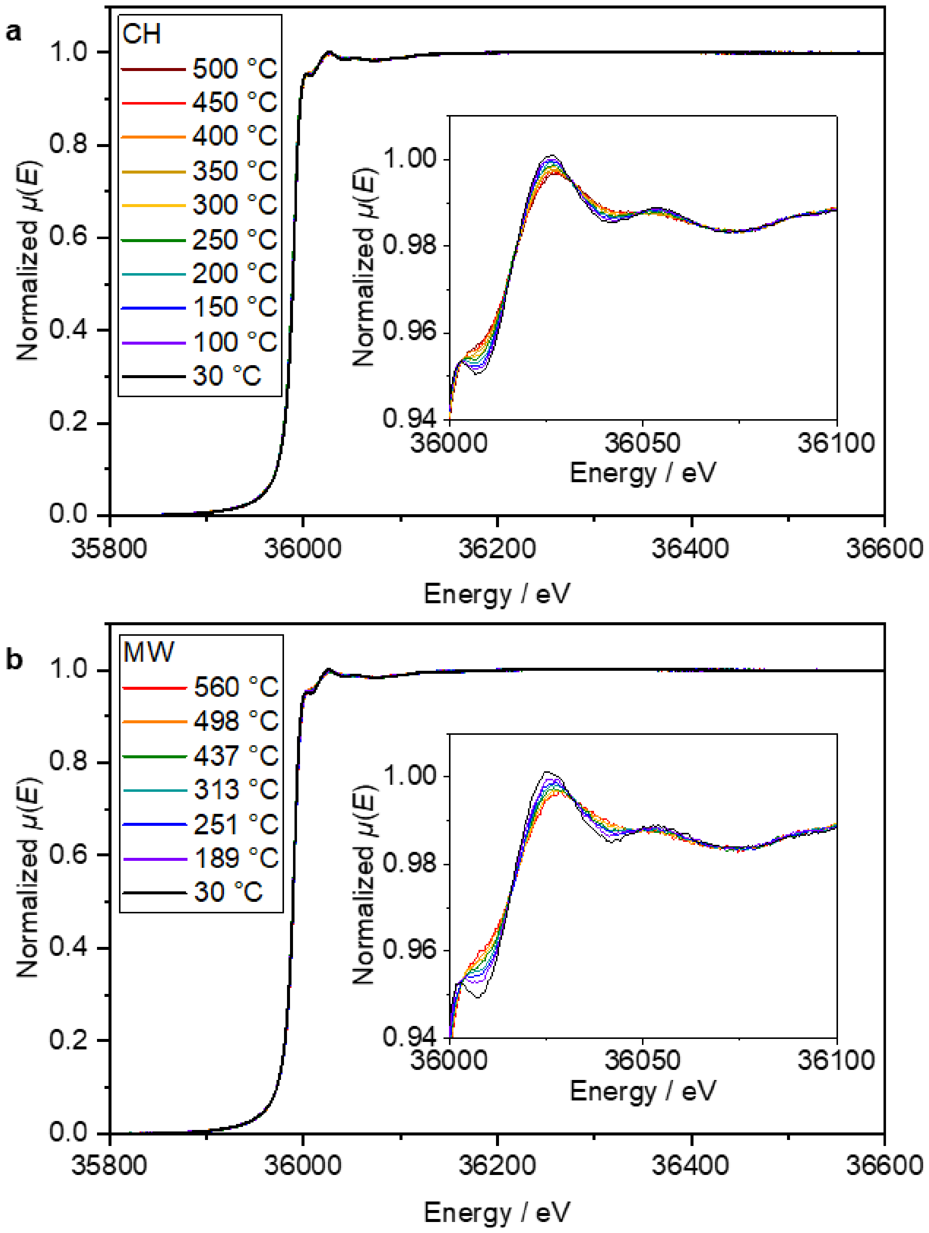

Fig. S37| Cs K-edge X-ray absorption near-edge structure (XANES) of Cs^{+}-FAU under MWs or CH.

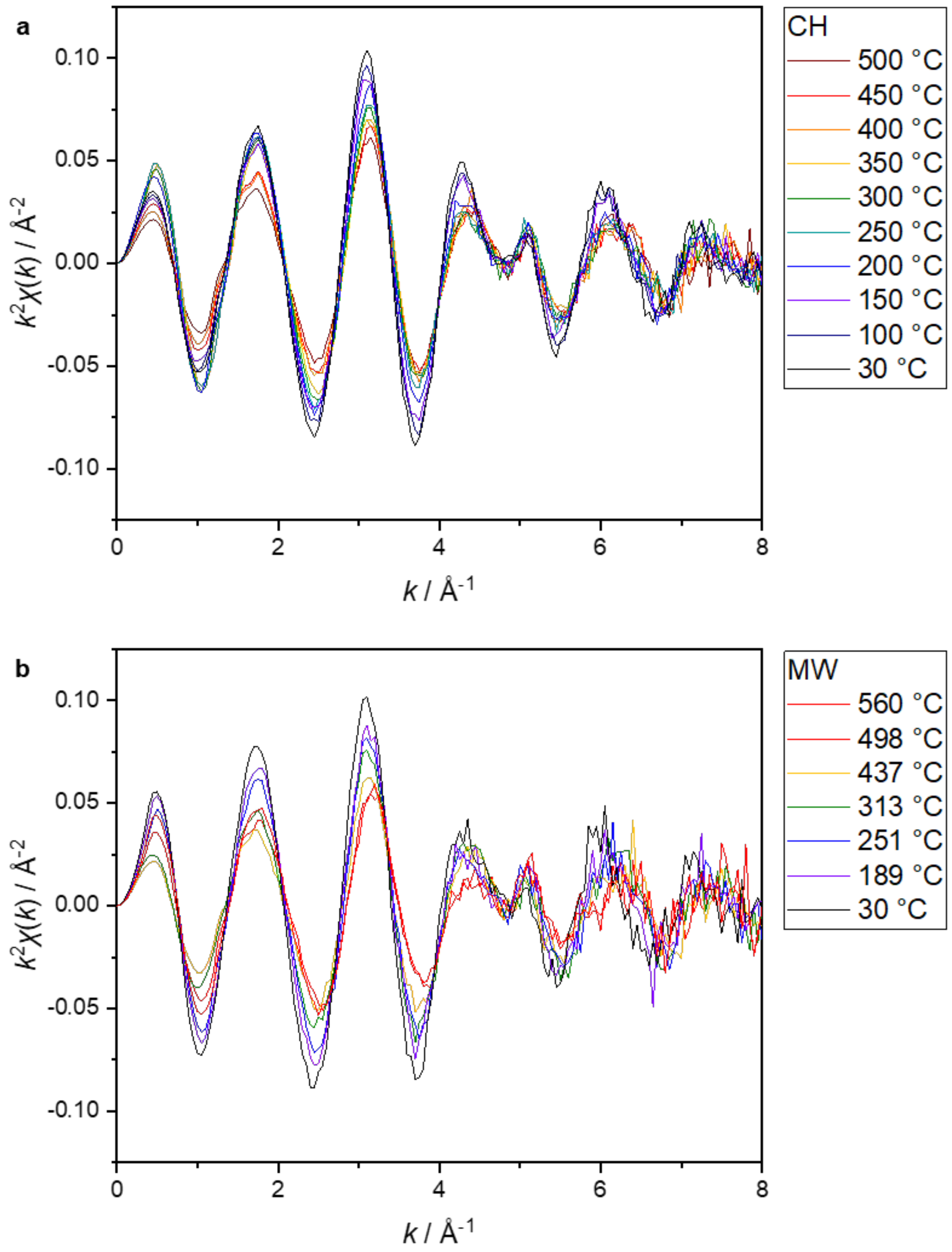
a
0.10
0.05
0.00
-0.05
-0.10
$k^2\chi(k)$ / Å^{-2}
0
2
4
6
8
k / Å^{-1}
CH
500 °C
450 °C
400 °C
350 °C
300 °C
250 °C
200 °C
150 °C
100 °C
30 °C
b
0.10
0.05
0.00
-0.05
-0.10
$k^2\chi(k)$ / Å^{-2}
0
2
4
6
8
k / Å^{-1}
MW
560 °C
498 °C
437 °C
313 °C
251 °C
189 °C
30 °C

Fig. S38| k^2-weighted Cs K-edge extended X-ray absorption fine structure (EXAFS) of Cs^+-FAU under MWs or CH.

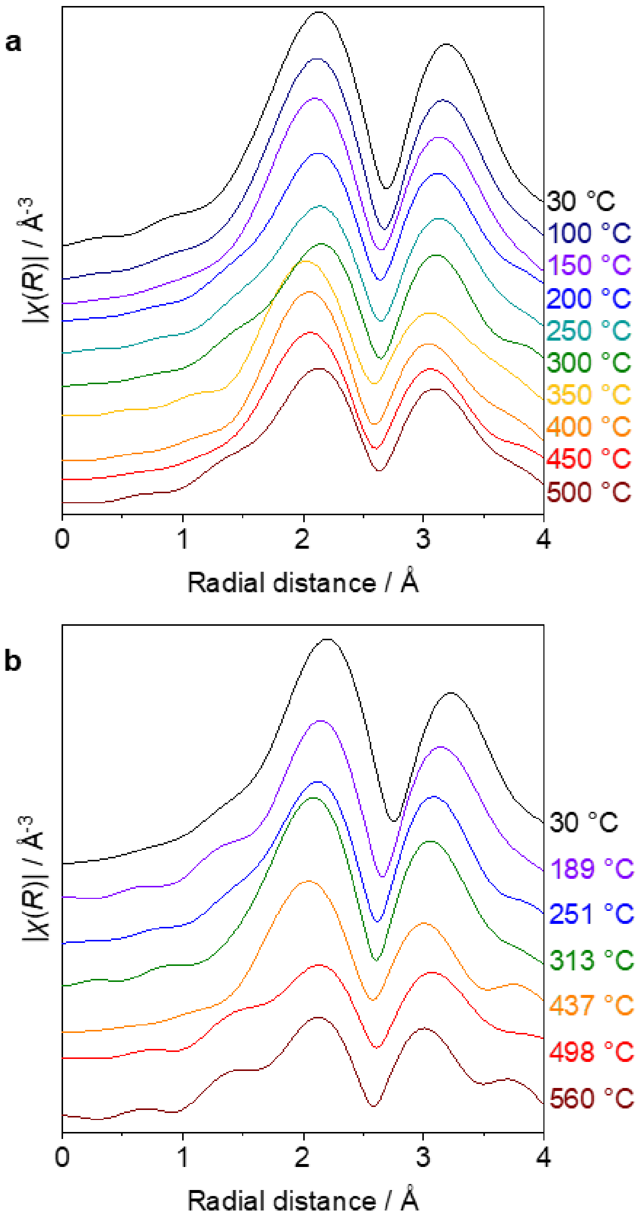

Fig. S39| k^2-weighted Cs K-edge Fourier-transformed extended X-ray absorption fine structure (FT-EXAFS) of Cs^+-FAU under MWs or CH.

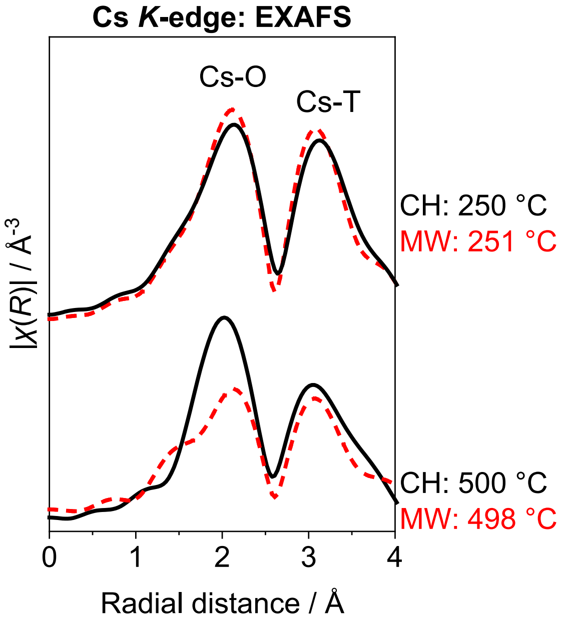

Fig. S40| Comparison of k^2 weighted Cs K–edge FT–EXAFS results of Cs^+–FAU under CH and MW.

Table S1. Dielectric property at 900 – 930 MHz of alkali metal cation exchanged zeolites

	As synthesized (hydrated), measured at 25 °C		Dehydrated at 500 °C, measured at 25 °C under dried N_2		Measured at 500 °C under dried N_2	
	**Upper: ε"**<br>**Lower: ε'**	**Upper: tanδ**<br>**Lower: σ^a**	**Upper: ε"**<br>**Lower: ε'**	**Upper: tanδ**<br>**Lower: σ^a**	**Upper: ε"**<br>**Lower: ε'**	**Upper: tanδ**<br>**Lower: σ^a**
**Cs-FAU**	0.410	0.244	0.189	0.073	2.187	0.439
	1.682	0.021	2.592	0.010	4.982	0.111
**Rb-FAU**	1.055	0.190	0.003	0.001	0.559	0.137
	5.551	0.054	5.551	0.003	4.071	0.028
**K-FAU**	0.989	0.124	0.112	0.017	0.617	0.111
	8.000	0.050	6.462	0.006	5.551	0.031
**Na-FAU**	1.172	0.132	0.157	0.028	n.t.	n.t.
	8.855	0.059	5.551	0.008		

n.t. = not tested

[a] ionic conductivity, σ / S m^{-1}, calculated from the following equation;

$$\sigma = \omega \varepsilon_0 \varepsilon' \tan \delta$$

where, ω / s^{-1} is an angular frequency of the incident microwaves, ε_0 is a permittivity of vacuum (8.854 × 10^{-12} / F m^{-1}), and ε' is relative permittivity of the material.

Table S2. Elemental composition of alkali metal cation exchanged zeolites

	**Si/Al**[a]	**Na/Al**[a]	**Cs/Al**[b]	**K/Al**[b]
**Cs-FAU**	2.8	0.02	0.67	-
**Rb-FAU**	n.t.	n.t.	-	-
**K-FAU**	2.8	trace	-	0.37
**Na-FAU**	2.8	1.0	-	-

[a] measured by ICP-OES

[b] measured by AAS

Movie S1. **Molecular dynamics simulation movie of Cs^+-FAU zeolite under conventional heating (within 200 ps).**

Movie S2. **Molecular dynamics simulation movie of Cs^+-FAU zeolite under microwave heating (within 200 ps)**

Movie S3. **Molecular dynamics simulation movie of Na^+-FAU zeolite under conventional heating (within 200 ps)**

Movie S4. **Molecular dynamics simulation movie of Na^+-FAU zeolite under microwave heating (within 200 ps)**
